# Development of an image classification pipeline for atherosclerotic plaques assessment using supervised machine learning

**DOI:** 10.1186/s12859-022-05059-1

**Published:** 2022-12-14

**Authors:** Natasha N. Kunchur, Leila B. Mostaço-Guidolin

**Affiliations:** Department of Systems and Computer Engineering, Carleton University, Ottawa, Canada

**Keywords:** Coherent anti-stokes Raman scattering (CARS), Atherosclerosis, Supervised machine learning, Support vector machine, Decision tree, K-nearest neighbour (kNN), Otsu thresholding, Marker-controlled watershed, K-means segmentation, Coefficient of variation feature selection, Filter-type feature selection

## Abstract

**Background:**

During atherosclerosis, the narrowing of the arterial lumen is observed through the accumulation of bio compounds and the formation of plaque within artery walls. A non-linear optical imaging modality (NLOM), coherent anti-stokes Raman scattering (CARS) microscopy, can be used to image lipid-rich structures commonly found in atherosclerotic plaques. By matching the lipid’s molecular vibrational frequencies (CH bonds), it is possible to map the accumulation of lipid-rich structures without the need for exogenous labelling and/or processing of the samples. CARS allows for the visualization of the morphological features of plaque. In combination with supervised machine learning, CARS imaged morphological features can be used to characterize the progression of atherosclerotic plaques.

**Results:**

Based on a set of label-free CARS images of atherosclerotic plaques (i.e. foam cell clusters) from a Watanabe heritable hyperlipidemic rabbit model, we developed an automated pipeline to classify atherosclerotic lesions based on their major morphological features. Our method uses image preprocessing to first improve the quality of the CARS-imaged plaque, followed by the segmentation of the plaque using Otsu thresholding, marker-controlled watershed, K-means segmentation and a novel independent foam cell thresholding segmentation. To define relevant morphological features, 27 quantitative features were extracted and further refined by a novel coefficient of variation feature refinement method in accordance with filter-type feature selection. Refined morphological features were supplied into three supervised machine learning algorithms; K-nearest neighbour, support vector machine and decision tree classifier. The classification pipeline showcased the ability to exploit relevant plaque morphological features to accurately classify 3 pre-defined stages of atherosclerosis: early fatty streak development (EFS) and advancing atheroma (AA) with a greater than 85% class accuracy

**Conclusions:**

Through the combination of CARS microscopy and computational methods, a powerful classification tool was developed to identify the progression of atherosclerotic plaque in an automated manner. Using a curated dataset, the classification pipeline demonstrated the ability to differentiate between EFS, EF and AA. Thus, presenting the opportunity to classify the onset of atherosclerosis at an earlier stage of development

**Supplementary Information:**

The online version contains supplementary material available at 10.1186/s12859-022-05059-1.

## Introduction

### Atherosclerosis: a deadly and silent disease

Atherosclerosis is a progressive disease, with a characteristic increase in lipid accumulation, inflammation, and plaque formation within the arterial walls [1]. As a chronic inflammatory condition, one of its hallmarks is the narrowing of the arterial lumen through plaque buildup within the inner lining of artery walls. Atherosclerotic plaques eventually lead to the blockage of blood flow, causing in extreme cases, the eventual rupture of the arteries [2].

**Fig. 1:**
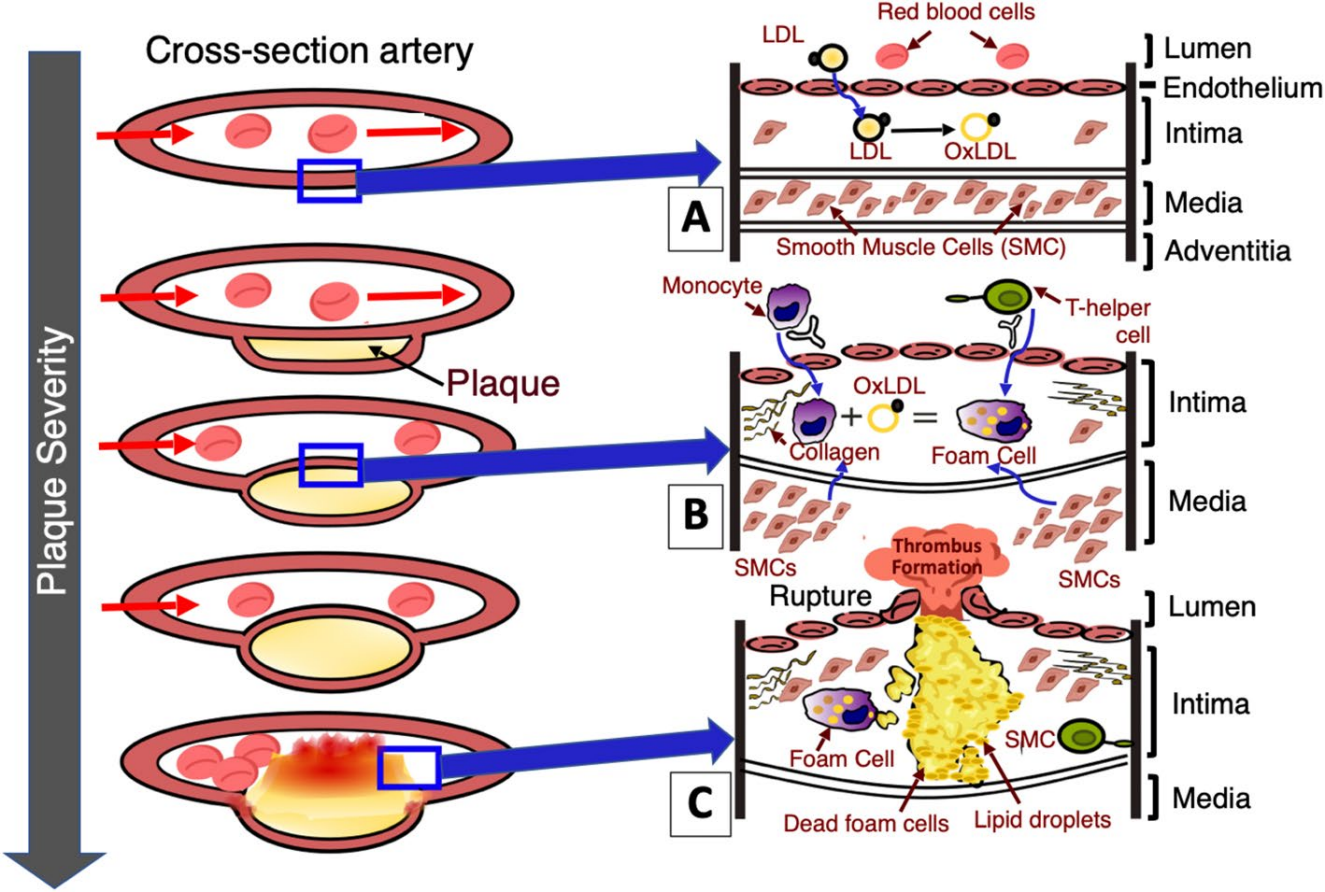
Schematic of atherosclerotic plaque progression in arteries. **A** Low-density lipoprotein (LDL) deposits in the tunica intima, becoming oxidized, activating endothelial cells. **B** Adhesion of leukocytes to activated endothelium cells allows monocytes to migrate into the tunica intima layer of artery, becoming macrophages. Macrophages absorb oxidized LDL, forming foam cells. Foam cells promote the migration of smooth muscle cells (SMC) from the media into the tunica intima, while simultaneously promoting smooth muscle proliferation. Increase in smooth muscle proliferation increases collagen synthesis, leading to the hardening of the atherosclerotic plaque. Foam cell death releases foam cell lipid content, driving the proliferation of plaque. **C** Increased plaque growth, increases pressure within the intima, ultimately causing tearing and rupture of the artery. Rupture leads to thrombus formation, impeding blood flow. Image adapted from [8], with permission from author [Armando Hasudungan]

Damage caused by hyperlipidemia, hypertension, enovironmental allergens, smoking and other noxious agents, disrupts the homeostatic condition of the artery wall. In turn, it will induce an excessive fibroproliferative response to repair injured and/or damaged epithelium and smooth muscle [3]. The atherosclerotic lesions promote an intracellular signaling cascade, highlighted in Fig. 1. It involves interactions between oxidized low-density lipoprotein (LDL) deposits, endothelial cells, leukocytes, monocytes, macrophages, T-lymphocytes and smooth muscle cells, all of which are involved in promoting cellular migration and proliferation to drive the formation of foam cell clusters abundant in atherosclerotic plaque [4].

The severity of the disease is often observed to increase with age. Symptoms tend to manifest when the lesions are already advanced and prone to cause significant consequences such as stroke or even death [5]. In the initial phase of plaque burden development, minimal build-up of plaque is observed in the intima of the artery, allowing the arterial lumen to remain unaffected. As the plaque expands, remodelling is observed to push the vessel wall outwards to maintain the volume of the arterial lumen. The formation of plaque build-up within the intima provides some indicators into the severity of the disease. Rapid growth of plaque development caused by increased lipid deposition are prone to rupture. The presence of a large lipid core with a thin fibrous cap places strain on the intima, ultimately leading to rupture and the potential onset of acute thrombosis [6]. In contrast, with a steady expansion of plaque, there is a lower risk of rupture, allowing the fibrous cap to remain intact in the presence of lower lipid deposition [7].

### Shining light into arteries: visualizing lipid-rich structures of atherosclerotic plaques

The visualization of atherosclerotic lesions has proven to be invaluable in clinic and fundamental research. Conventional clinical methods currently used to detect atherosclerosis lack sensitivity in detecting plaque morphology and its biochemical composition. Medical imaging modalities commonly used to present information about stenosis, rupture potential, thickness of the fibrous cap and the necrotic core include intravascular ultrasound (IVUS), optical computed tomography (OCT), positron emission tomography (PET), and magnetic resonance imaging (MRI) [9–14]. Though these established clinical imaging techniques provide valuable information to diagnose developed atherosclerosis, they present limited capability in characterizing the unique plaque compositions contained within the intima of the arterial wall. The aforementioned imaging modalities lack detailing morphology, and biological features unique to the biochemical composition of plaques [15]. Said insight is crucial in unveiling fundamental mechanisms associated with the disease progression, the effects of novel therapies, identifying the severity of the disease and the potential impact of diet on macrophage infiltration in the arterial lumen [16].

Provided that the evolution of macrophage infiltration, lipid-laden foam cell accumulation, extracellular lipid distribution and fibrous tissue deposition all contribute to defining plaque morphology, it is necessary to use an imaging modality capable of capturing these distinct plaque attributes. The listed biomolecular features provide greater insight into the progression of atherosclerosis and can be imaged non-invasively by a non-linear optical modality, coherent anti-stokes Raman scattering (CARS) microscopy [17]. CARS allows for the acquisition of images with high spatial resolution and high biochemical specificity, without the use of exogenous labels and additional sample preparation [18]. The abundance of CH bonds in lipid-rich structures present in atherosclerotic plaque exhibit distinct vibrational signatures. To date, CARS microscopy has been extensively used to characterize lipid-rich structures in biological tissues, successfully revealing the organizational structure of arterial vessel membranes and capturing drastic contrast in the appearance of healthy tissue and plaque-containing tissue [19–24]. Applied within *ex-vivo* environments, multiplex CARS has proven to image intact morphologies of atherosclerotic lipids with correlative chemical information. Demonstrated by Kim et al. [25], the progression of lipid accumulation within arteries was spatially analyzed using 3D CARS imaging. Provided that atherosclerosis can be largely prevented by following a healthy lifestyle, understanding how external factors such as high-fat diets and noxious agents can trigger the onset of the disease is critical. Using an animal model, Lim et al. [16] revealed that by following a high fat, high-cholesterol Western diet resulted in an approximate twofold increase in intimal plaque area, defined by CARS signals of lipid-rich macrophages. Application of CARS is not only limited to the quantitative assessment of dietary impact in early-stage atherosclerotic plaque progression, it can further differentiate the stage to which the disease has progressed by successfully analyzing plaque composition [26]. Thus far, research has cohesively showcased CARS potential in leading investigations of lipid-driven disorders and respective preclinical drug screenings. Despite the biochemical specificity provided by CARS microscopy and its potential applications, there has been, however, limited efforts to extract relevant plaque morphological features to be used as a basis to classify these label-free images.

### Supervised machine learning to classify plaque burden: our contribution

CARS imaging has the capability to extract morphological features which can be correlated to biological indicators unique to the varying atherosclerotic lesion progressed disease states. However, the full potential of CARS to automatically identify and classify lesions based on their morphological details has not been yet been fully explored. With non-invasive imaging modalities becoming instrumental in visualizing atherosclerosis, there has been a growing demand for efficient pattern recognition tools to discriminate amongst imaged tissue structures. Supervised machine learning (ML) algorithms have proven to be strong pattern recognition tools, classifying minute biological details to high degrees of accuracy [27]. Used in accordance with CARS, supervised machine learning models can provide higher sensitivity in capturing minute morphological changes during the progression of atherosclerosis; changes which can often be overlooked by the human eye.

Supervised machine learning applies labeled data derived experimentally to fit a classification model [28]. When paired with imaging modalities, supervised machine learning algorithms have been successfully employed to improve diagnostic performance by demonstrating maximal discriminatory abilities [29]. Powerful and efficient supervised machine algorithms include support vector machines, k-nearest neighbour classifiers and decision trees. Using hyperplanes to discriminate amongst classes and their respective features, support vector machine (SVM) models were used to gain an accurate assessment of myocardial perfusion by localizing the mitral valve plane during image acquisition [30]. By training the model on approximately 400 unique scans, the SVM classifier outperformed classification performance of two practicing radiologists when detecting obstructive coronary artery disease, with respective AUC scores of 0.82 for the SVM model and 0.79 and 0.81 AUC scores of the experts.

Often defined as the most intuitive learning algorithm, k-nearest neighbours (kNN) work by assigning target labels derived from those of its *k* nearest neighbors (determined according to a distance measure computed from the inputs) within a learning sample [31]. Though simple in nature, Takx et al. [32] performed two-stage classification with nearest neighbour and SVM classifiers for the automation of coronary artery calcium (CAC) scoring using imaged scans of the chest. The developed approach resulted in acceptable reliability in comparison to a manually determined reference standard for CAC scoring. Similar results were obtained by Kang et al. [33] utilizing an SVM to automatedly detect non-obstructive and obstructive coronary artery disease with a high accuracy of 94%.

Decision trees, valued for their efficiency and ability in capturing nonlinear relationships, have been used to identify pulmonary hypertension (PH) without the requirement of invasive right heart catheterization [34]. Image-based metrics of PH scans improved diagnostic accuracy by leveraging qualitative descriptors from scans of the main pulmonary artery. The decision tree model demonstrated the ability to non-invasively diagnose PH with high accuracy (92%) using computation-derived metrics capturing hemodynamic changes in the pulmonary vasculature with measurement of right ventricular morphology and function.

Unique to supervised machine learning, features relevant to the input data are supplied for classification. The selection of these features can greatly affect the resulting classification performance metrics, either positively or negatively impacting the expected output. Commonly coupled with supervised machine learning are pre-processing feature reduction techniques aimed at eliminating irrelevant features used for classification. Conventionally, only features with high predictive power are selected, thus removing features deemed not valuable in aiding classification. However, as identified by Chakraborty et al. [35], removal of entire features may result in pertinent information being lost, negatively impacting the classification. Their work suggests that a differentiation between irrelevant and unimportant features must be made, as the removal of unimportant features is preferable in preserving key classification information. Hence, our work leverages two methods for feature refinement to differentiate amongst the relevancy and importance of features; a novel coefficient of variation (CV) method and a filter-type feature (FTF) selection method employing chi-square testing. The extracted features are quantitatively assessed, observing if the refined features are indeed important and able to capture variation amongst the three stages of atherosclerotic plaque progression (early fatty streak development, early fibroatheroma, advancing atheroma).

Despite the high level of detail observed within CARS-imaged atherosclerotic plaque, minute morphological indicators unique to plaque progression can easily be overlooked. The main contribution of our work is to present an automated image classification pipeline able to attribute complex lesion characteristics with the corresponding progressed stage of atherosclerosis. By successfully combining label-free imaging with a robust method for feature extraction, this ex-vivo study presents a novel classification methodology able to differentiate amongst the three major stages (EFS, EF and AA) of atherosclerosis. Using a novel feature refining methodology; first-order statistics, shape and textural features with high predictive capabilities are selected as defining metrics associated with lipid-rich structure accumulation. Thus, tracking the morphological changes amongst EFS, EF and AA to a high degree of accuracy. The proposed pipeline’s performance is evaluated using varying evaluation metrics, quantitatively assessing the accuracy of the plaque segmentation, value of the extracted features and classification performance of the supervised ML algorithms. Through the development and application of this novel pipeline, we are capable of rapidly surveying diseased bulk arterial tissue.

This paper provides a detailed overview on the development of the novel automated classification pipeline. The curation of our dataset and the methodology followed is provided; defining the animal model selected, sample preparation and the specifics of applied CARS microscopy. The individual methods used to develop our pipeline are also outlined, providing insight into the various image preprocessing, image segmentation, feature extraction, feature refinement and supervised ML algorithms applied and tested. Results provide the performance metrics obtained from the classification pipeline and the combination of methods providing high levels of accuracy. We discuss how these varying tools can be applied in unison to achieve a greater degree of accuracy when classifying the stages of atherosclerotic plaque progression. Not only is a comparative analysis amongst image processing, feature refinement methods and supervised ML techniques conducted but the specific (first order, shape and textural) features used and their effectiveness in quantitatively assessing plaque morphology is evaluated. Lastly, the scope of future work is discussed, followed by conclusions and the applicability of our work in assessing atherosclerotic plaques.

## Materials and methods

### Animal model and tissue preparation

Animal experiments were performed in accordance with protocols approved by the local Animal Care Committee of the former Institute for Biodiagnostics at the National Research Council of Canada in Winnipeg (Project IBD2006.16-ACC). A myocardial infarction-prone Watanabe heritable hyperlipidemic (WHHLMI) rabbit model was used. Through selective breeding, WHHLMI rabbits develop a hereditary defect in low-density lipoprotein (LDL) processing and become prone to atherosclerosis onset without the requirement of a modified diet [36]. Arterial samples were harvested from a total of twenty one WHHLMI rabbits aged between 0 and 27 months. This span of pre-defined age groups is aimed to model the progression of atherosclerosis and its corresponding stages. Twenty seven months old is the end of the diseased rabbits life cycles. The aortas were dissected from the ascending aorta to the external iliac artery. Upon excision, they were rinsed in heparnized saline. The exterior aorta, subdivided into 60–80 mm sections, were cut open longitudinally to expose the luminal surface. The hydration of the samples was maintained by applying PBS solution periodically. It was further ensured that the luminal surface remained facing up on a moist surface.

### CARS microscopy imaging

A custom-built multimodal NLO laser scanning microscope, previously described elsewhere was used for CARS imaging of arterial tissues [37]. In short, the light source was generated by a Ti:Sapphire oscillator (Spectra-Physics, Tsunami) with a center wavelength at 800 nm, a pulse width of 100 fs and integrated with a photonic crystal fiber (PCF, add manufacturer). The near infrared (NIR) portion (900 nm) of the broadband emission was used as the Stokes beam for CARS imaging, while the beam generated directly by the Ti:Sapphire oscillator was used as a pump pulse. The pump and Stokes pulses were combined at a beam combiner before proceeding into the laser scanning microscope assembly. A ×20, 0.75 NA infinity corrected air objective lens (Olympus Canada, Markham, ON, Canada) focused the laser pulses on samples and collected the epi-CARS signal. Typically, 25 mW of pump and 8mW of Stokes (measured after the ×20 air objective lens) were used for imaging. A photomultiplier (PMT) detector (Hamamatsu) was used for epi and forward direction signal detection of the filtered laser pulses. ScanImage (ver. 3.5) software was used for image acquisition and laser scanning control. Pixel dwell time for an average of 4 scans for a single collection was 21 μs.

### Stages of atherosclerotic plaques

Based on the American Heart Association (AHA), the major distinctions in the development of atherosclerotic plaque can be classified into four primary classes: early fatty streak (EFS) development, early fibroatheroma (EF), advancing atheroma (AA) and complex lesion (CL) development [38]. The presented histological and morphological classification of atherosclerotic lesions, as described in Table 1, provides a standard framework to correlate the composition of lesions to the clinical manifestation of the disease.

**Table 1:** Stages of atherosclerosis and their respective morphologies

Stage	Descriptors
Type I and II Early fatty streak (EFS) development	First observed when LDL departs the bloodstream to cross into the arterial intima Oxidation of LDL in the intima promotes a cascade of proinflammatory cells and proteins, invoking an inflammatory response within the arterial lining Active smooth muscle cell secretion of chemokines and chemoattractants promotes monocyte migration and monocyte transformation into macrophage migration Lipid intake by macrophages drives plaque formation with lipid accumulation developing as confluent extracellular lipid pools with decreased cellularity EFS is most often observed in childhood and adolescence [24, 25]
Type III Early fibroatheroma (EF)	Marks the bridge between early and advanced lesions, classified as the most difficult stage to identify Excessive uncontrolled inflammatory response exaggerates macrophage accumulation leading to increased accumulation of large pools of lipid coalesces and cell necrosis A combination of enlarged lipid pools and necrosis distorts the architecture of the intima Early fibroatheroma is the first stage to observe the formation of the fibrous cap over necrotic cores to form dominant lesions occupying thirty to fifty percent of the arterial wall volume EF is observed to occur after puberty [24, 25]
Type IV Advancing atheroma (AA)	First lesion to be considered advanced by histological criteria and is used as an umbrella term encompassing all lesions that disrupt intimal structures Unchecked activity of proteolytic enzymes dissolves fibrous tissue, thinning the fibrous cap surrounding the necrotic core Severe disarrangement of intimal structure is caused solely via extensive accumulation of extracellular lipid localized in the deep intima Dense accumulation of extracellular lipid occupies a dominant region of the intima AA is on average observed in ages 55 plus [24, 25]
Type V and VI Complex lesion (CL) development in advancing atheroma	Development of complex lesions, classified as Type V and VI lesions are abstract in formation, deviating from the simple accumulation of lipids observed in EFS and EF Type V lesions are thickened by reparative collagenous tissue layers Large accumulations of extracellular lipids disarrange the normal cell and intercellular matrix structure Subtypes Va and Vb of type V lesions can be further differentiated respectively by the presence of fibrous connective tissue and calcific lesion Type VI reflects surface defects, hematoma and thrombotic deposits The four subtypes of lesion VI include differences in the composition of the blood, relative quantities and distributions in the components of the underlying lesion, and the modifications of shear and tensile forces to which the lesion or intima is exposed [24, 25]

For this work, complex lesions within Advancing Atheroma (Type V and VI) were excluded as the development of collagenous tissue layers, and thrombosis were not assessed using CARS imaging. Complex lesions (type V and VI) fall within the umbrella of advancing atheroma, with the intended purpose of this work to differentiate and identify between early stage and advanced stage atherosclerosis.

### Dataset and types of lesions evaluated

**Fig. 2:**
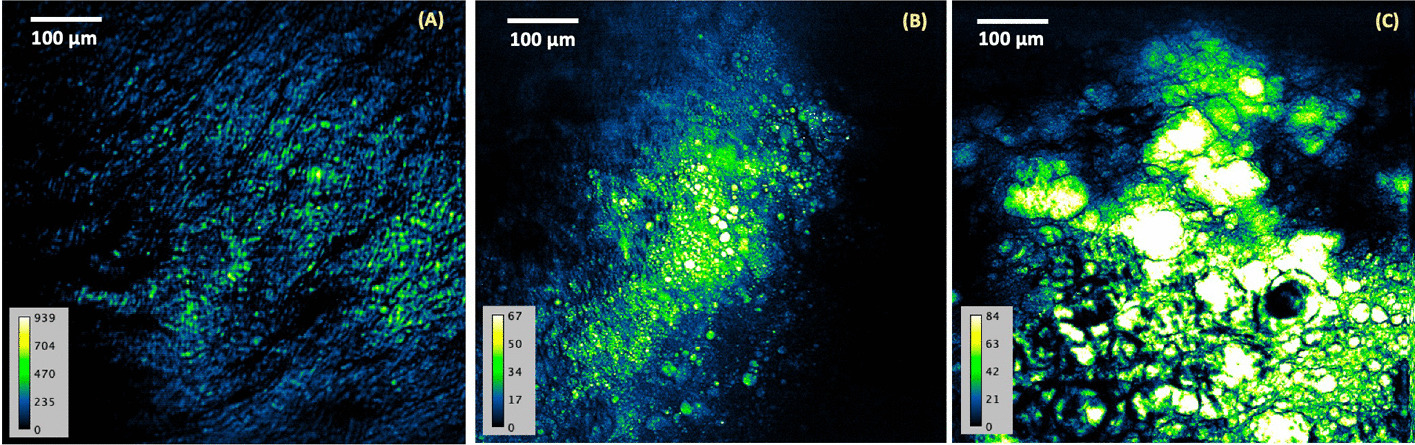
Representative CARS image acquired from luminal surface of the WHHLMI rabbit artery, that is **A** 6 months-old, **B** 14 months-old, **C** 27 months-old. (×20 air objective lens, 0.75 NA). Pseudo-colour code: blue (lower density of foam cells)/yellow (higher density of foam cells). Colour bar represents the density of foam cells with lighter colour equating to higher densities of foam cells commonly observed in older and structured plaque

In this ex-vivo study, a total of 566 CARS images were used to train an automated classification pipeline correlating first order statistics (FOS) features, shape features and intensity-based parameters to atherosclerotic plaque burden. Atherosclerosis is a progressive disease and the severity and progression of the disease are often dependent on age. Using the age of the WHHLMI rabbit model as a guiding factor, the expected temporal pattern of plaque accumulation unique to the respective morphologies of EFS, EF and AA can be identified. To curate a training, test and validation set, the 566 images were initially manually segregated into their respective disease severity stages (EFS, EF and AA). Following the visual and biochemical descriptors identified in Table 1, the CARS imaged plaque was assembled as Group EFS (214 images), Group EF (134 images) and Group AA (218 images). Representative CARS plaque from the three classes, EFS, EF and AA heart are illustrated in Fig. 2 in that respective order. The observations are consistent with the morphological features described in Table 1. As the disease progresses, there is a significant accumulation in lipid deposits with initial streaks of fat observed in EFS developing into more defined lipid coalesces characteristic of advanced atheroma. The CARS images provide a high level of detail on forming lipid-rich structures. Thus, allowing clear distinctions to be made about the effects of the disease in atherosclerotic arteries of the WHHLMI rabbit model.

### CARS images classification pipeline

**Fig. 3:**
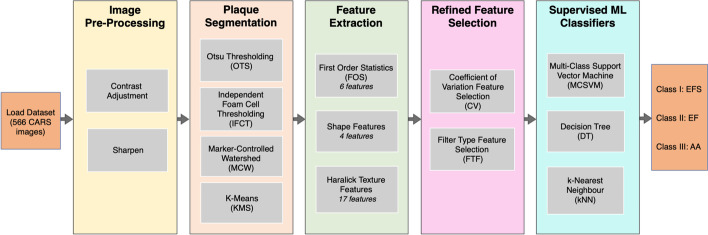
Automated classification pipeline architecture. CARS atherosclerotic plaque images are input to the first image preprocessing stage, undergoing contrast adjustment and sharpening. Output of image preprocessing for all 566 images is fed into the four segmentation methods, OTS, IFCT, MCW and KMS to isolate the lipid-rich structures of the plaques. Features with high predictive power are computed from the isolated outputs. Refined features are fed into the four supervised ML algorithms, MCSVM, DT and KNN to output the classification of atherosclerotic plaque severity as early fatty streak development (EFS), early fibroatheroma (EF) and advancing atheroma (AA)

Differences in signal associated with foam cells (lipid-filled macrophages) of the imaged plaque were classified using our developed automated classification pipeline, shown in Fig. 3. The pipeline is composed explicitly of five steps, commencing with the pre-processing of the entire dataset using contrast adjustment and sharpening to increase the quality of the CARS-imaged atherosclerotic plaque. Following image preprocessing, four image segmentation methods are employed in parallel. Comparisons of the outputted segmentations are statistically assessed, identifying the method producing the most consistent and accurate plaque boundary segmentations. From the dataset of segmented plaques, a total of twenty-seven feature parameters inclusive of FOS, shape, and textures are extracted for each segmented plaque and further refined using coefficient of variation (CV) method and FTF selection method. Lastly, the refined features are supplied into three supervised machine learning classification algorithms in parallel, testing which framework (image segmentation method, refined features and supervised ML algorithm) provides the best performance metrics in differentiating amongst the three stages of plaque progression (EFS, EF and AA). All 566 images are simultaneously loaded into the pipeline, outputting the classification of the disease stage based on the segmented plaque and its extracted refined features.

### Image pre-processing, segmentation and performance assessment

A total of four image segmentation methods were tested to isolate the lipid-rich structures of the plaques. All images were first enhanced using in-built Matlab contrast adjustment and image sharpening functions before segmentation. Contrast adjustment and image sharpening both operate by automatically limiting the range of pixel intensity values of respective image histograms to the dominant pixel intensities captured within the middle portion of the range. Otsu Thresholding, Independent Foam Cell Thresholding, Marker Controlled Watershed and K-Means segmentation were applied to segment the image plaque from its surrounding backgrounds. All 566 images were independently fed into each segmentation method to identify the method capable of segmenting plaque with the highest degree of accuracy. A brief description of each segmentation method used is provided in Additional file 1: Table S1.

**Fig. 4:**
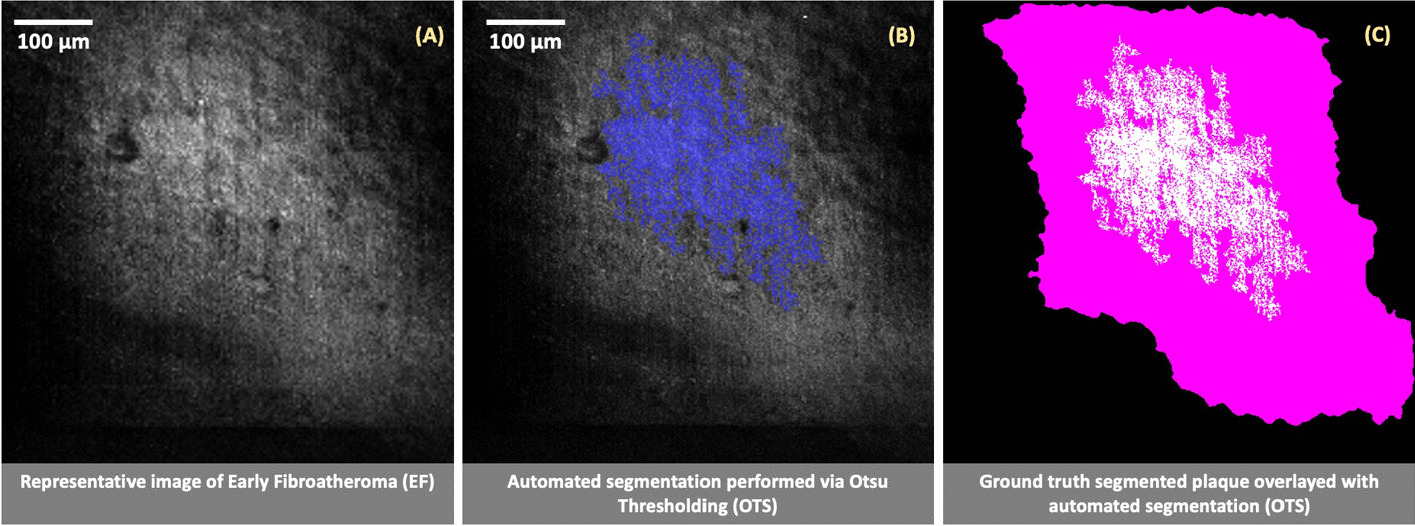
Evaluation of plaque segmentation methods shown using a representative image (**A**) belonging to early fibroatheroma and its respective automated segmentation performed using Otsu thresholding (**B**). Ground truth of the isolated plaque is acquired using active contours to manually trace the boundary of the plaque (highlighed in pink), overlaying it with the automated segmentation performed by OTS (**C**) to calculate a Dice coefficient of 0.52

To classify lipid-rich structures, accurate segmentation of foam cell clusters is required. Dice coefficient was applied to a random subset of fifty images per group, inclusive of images from all three diseased states (EFS, EF and AA) [39]. The ground truth image was curated manually by employing active contours boundary tracing [40]. To segment the plaque specifically, an initial mask unique to each plaque’s boundaries was defined, specifying the initial state of the active contour (Fig. 4).

### Feature extraction

For each segmentation method; textural, shape and FOS features were extracted from the isolated (segmented) plaque. A total of twenty-seven features were defined from each isolated plaque with six features defining FOS, four features defining shape and seventeen features defining the textural elements of the plaque morphology. FOS is related to the frequency of occurrence of a gray tone within an investigated region. Extracted FOS features included mean intensity, number of lipid cells, skewness, kurtosis, standard deviation and integrated density. Shape features provide visual descriptors of the imaged plaque, quantifying the area, circularity, perimeter and extent of the isolated plaque. A total of seventeen textural features, originally defined by Haralick et al. [41] were calculated based on the image histogram and the gray level co-occurrence matrix (GLCM) of the image. The GLCM represents the probability of occurrence of a pixel pair holding a given gray-tone difference separated by a predefined distance and direction. The image histogram counts the frequency of occurrence of the gray tone pair within a region of interest (ROI). The GLCM was calculated in four orientations: 0°, 45°, 90° and 135°. A computational window size of 9 pixels was adopted to extract features from 16-bit images. The values extracted from all four orientations were averaged. From the co-occurrence matrix, the following Haralick textural features were extracted: autocorrelation, cluster prominence, cluster shade, contrast, correlation, difference entropy, dissimilarity, energy, entropy, the measure of correlation, maximum probability, sum average, sum entropy, the sum of squares variance, sum variance, homogeneity. Image pre-processing, segmentation and the calculation of FOS, shape and textural feature parameters were performed using a custom-built image processing toolbox on MATLAB7.5, leveraging a few in-built MATLAB functionalities.

### Feature selection

To statistically capture interclass feature variation, a novel method utilizing the CV was implemented. The CV is the standard deviation of the mean. A higher value CV signifies a greater observed dispersion in relation to the mean [42]. For each disease state (i.e. EFS, EF and AA), the averages of each feature were taken. This process was completed independently for each of the four segmentation methods. If the set threshold of 8% for the CV was crossed, the feature demonstrated enough variance between the three classes, translating into higher feature predictive power.

To evaluate the robustness of CV and provide a comparative baseline, an automated FTF selection method was also applied independently. Within the domain of supervised machine learning, FTF is a commonly used method for feature refinement, selecting a subset of features scoring highly on their correlation with the outcome variable [43]. Wrapper methods and embedded methods were avoided as they are computationally expensive. Furthermore, feature selection in these two methods is completed by training independent models using subsets of features, either adding or removing features based on said model performance [43]. This in turn risks overfitting our pipeline as features unique to our CARS dataset would be selected. In contrast to wrapper and embedded methods, FTF selection evaluates the relationship between the input variable and target variable. Feature characteristics such as feature variance and feature relevance to response, provide a basis to filter input variables to be used by the model [44]. Each feature was ranked using chi-square tests by determining whether each predictor variable is independent of a response variable. The top fifteen ranked variables by FTF were selected as the refined features. Provided that the proposed CV feature refinement method evaluates feature importance statistically, FTF provides a closer baseline in conducting a comparative analysis of CV feature refinement and its selected features. Using FTF and CV, there is greater clarity behind the justification for selecting specific subsets of features. Whereas, with embedded and wrapper methods, we blindly rely on the model performance metrics when selecting subsets of features with high predictive power. In turn, this potentially harms the generalization capabilities of the selected features.

### Supervised ML classifiers

Supervised machine learning models have proven to be powerful tools for pattern recognition, applying externally supplied metrics to predict future instances [45]. The three supervised machine learning algorithms applied for atherosclerotic plaque morphology included multi-class support vector machine (MCSVM), decision tree (DT) and k-nearest neighbour (kNN) classifier; all implemented using the in-built MATLAB7.5 Mdl package. For each segmentation method, the three classifiers were independently trained, tested, and validated on the twenty-seven unique features characterizing each of the 566 isolated plaques. The MCSVM model trained is an error-correcting output codes (ECOC) classifier with a one-vs-one binary SVM design. This design exhausts all combinations of class pair assignments [46]. Bayesian optimization was performed to tune the hyperparameters of the ECOC-MCSVM model. Minimization of the cross-validation loss (error) by varying the hyperparameters of the ECOC-MCSVM model include but are not limited to: coding (one vs all, one vs one), box constraint, kernel scale, cache size, outlier fraction, or shrinkage period [47]. DTs are a robust supervised machine learning model popular for being non-parametric, greater resistant to noise and providing better generalization [45]. A fitted DT classifier optimized using Bayesian optimization hyperparameters was applied to classify the extracted features pertaining to plaque morphology. Cross-validation loss was minimized by optimizing leaf size, tree depth, number of splits and parent size. Lastly, a Bayesian optimized kNN classifier was used to assess the overlap in extracted features among morphologies. Classification by comparing CARS imaged plaque to their respective neighbours would highlight similarities and differences amongst the types of structures found in each of the three different plaque severity levels. The hyperparameters tuned to minimize cross-validation loss in the kNN include the number of neighbours used to vote on a decision and the distance metric used to calculate the proximity of neighbours. The train, test and validation sets were randomly defined using a respective 60/20/20 split. All classifiers were trained and validated using tenfold cross-validation.

## Results

### Evaluation of plaque segmentation

To identify the most accurate plaque segmentation method, a comparative analysis was conducted between OTS, IFCT, MCW, and KMS. It can be presumed that a higher segmentation accuracy will translate into stronger feature extraction and atherosclerotic plaque classification. The highest Dice coefficient observed belonged to OTS segmentation, scoring an averaged Dice index of 0.75, followed by an index of 0.63, 0.73 and 0.71 for IFCT, MCW and KMS respectively. A higher Dice coefficient equates to a higher segmentation accuracy. Initial image pre-processing increased the clarity of the imaged foam cells clusters, marking a stark contrast between the plaque and its surrounding areas. Formally, OTS functions by splitting an image into a foreground and background through the identification of an optimal gray-level threshold value. Hence, the exagerrated contrast between the high pixel intensities of plaque and the lower intensities of its surrounding areas provided a good foundation for OTS segmentation to find an optimal gray-level threshold. OTS further proved to be more sensitive at capturing sparse isolated foam cell clusters. The technique of IFCT segmentation is comparable to OTS, however, by individually segmenting foam cell clusters resulted in large overlapping areas of the plaque remaining unsegmented. Though MCW and KMS did not outperform OTS, the Dice indices noted the two segmentation methods to have worked relatively well. Using the image gradient to define ridges for the segmentation of plaque, enabled MCW to trace the complex boundaries of the foam cell clusters with a high degree of specificity. Lastly, supplying k-means with Gabor filters sensitive at detecting lipid arrangements allowed for the segmentation method to outperform IFCT.

### Initial poor performance metrics using all features

To identify the morphological characteristics unique to the three diseased states, feature extraction was applied. The first order statistics, shape descriptors and texture features for each of the isolated foam cell clusters were extracted. To test feature relevance and their predictor importance in classifying the disease state, all twenty-seven features were first employed into MCSVM, DT, kNN classifiers. The initial averaged classification accuracies outputted were limited, observing averaged accuracies amongst the three diseased states to be 60.51% for EFS, 56.77% for EF and 72.53% for AA. Additional file 1: Table S2 provides an overview of the initial observed accuracies, class precision and class recall scores from each segmentation method amongst the three classifiers.

kNN classification of atherosclerotic plaque segmented from OTS outperformed both MCSVM and DT classifiers using MCW, IFCT and KMS segmentation methods. As correctly presumed, a higher segmentation accuracy did translate into a higher initial classification accuracy. With an average segmentation accuracy index of 0.73, features extracted from OTS segmented foam cell clusters achieved a higher degree of classification accuracy. MCSVM showed to perform the worst amongst all classifiers, scoring exceptionally low F1 scores, reflecting poor class accuracies. The ECOC-MCSVM model uses a One vs One approach to reduce the multiclass classification problem by training binary learners for each pair of classes. The final class prediction is then performed by majority voting with the confidence criterion defined as the distance from the margin. A major shortcoming of this multi-classification approach is the surplus of support vector machines required to be trained. MCSVM works to identify a hyperplane that best separates between each pair of classes, neglecting features belonging to a third class. In this case, separation was maximized between EF and AA or EFS and EF, resulting in extremely low classification accuracies (approximately 30%) for the remaining third diseased state. With the exception of MCSVM, the classifiers on average performed best at classifying EFS and AA and struggled with the classification of EF diseased state. Given that early fibroatheroma is an intermediate stage between early fatty streak development and advancing atheroma, the morphology of EF hosts large similarities to the characteristics of both EFS and AA. A defining feature of early fibroatheroma is the excessive uncontrolled inflammatory response exaggerating macrophage accumulation. This increased accumulation of lipid coalesces into large pools distorts the architecture of the intima and is a phenomenon also common to advancing atheroma. Furthermore, an observed random network of macrophage deposits is a feature common to both EFS and EF.

The low-class accuracies, precision and recall scores outputted by the three classifiers indicate a case of poor generalization attributed to the model being overfit [48]. High generalization error scores were noted for each of the models, averaging approximately 0.45 for the three classifiers. Common methods to accommodate overfit models include removing outliers within the dataset, curating a larger dataset, removing class imbalance, and/or refining features [49]. Increasing the size of the dataset is not a feasible option for our study. Using data engineering methods, such as image mirroring and stretching to grow the dataset would negatively impact the morphologies unique to each foam cell clusters and could potentially lead to the model becoming more overfit. Class imbalance was not observed as the samples belonging to EFS, EF and AA contained 214 images, 134 images and 218 images respectively. Though outliers within the dataset may exist, it is not an appropriate approach to dismiss atherosclerotic lesions progressing outside of their norm. Therefore, all instances for atherosclerotic lesion development were accounted for.

### Refinement of features using CV and FTF

**Fig. 5:**
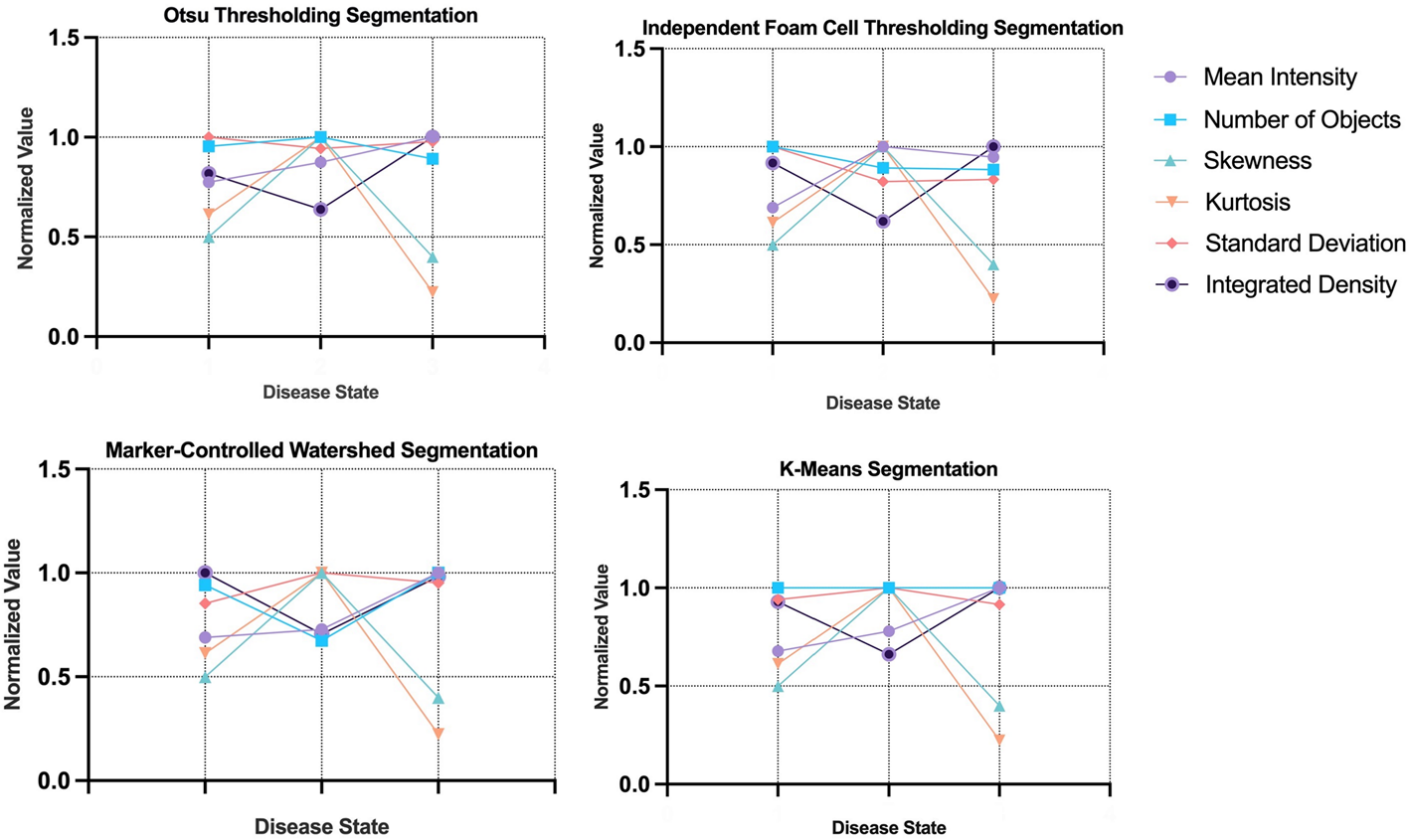
Comparison of variation in FOS features for each segmentation method, OTS, IFCT, MCW and KMS and the disease states of atherosclerosis, EFS, EF and AA. The variation is normalized using the maximum of each feature. FOS, first order statistic; OTS, otsu thresholding; IFCT, independent foam cell thresholding; MCW, marker-controlled watershed; KMS, k-means segmentation; EFS, early fatty streak development; EF, early fibroatheroma; AA, advancing atheroma

**Fig. 6:**
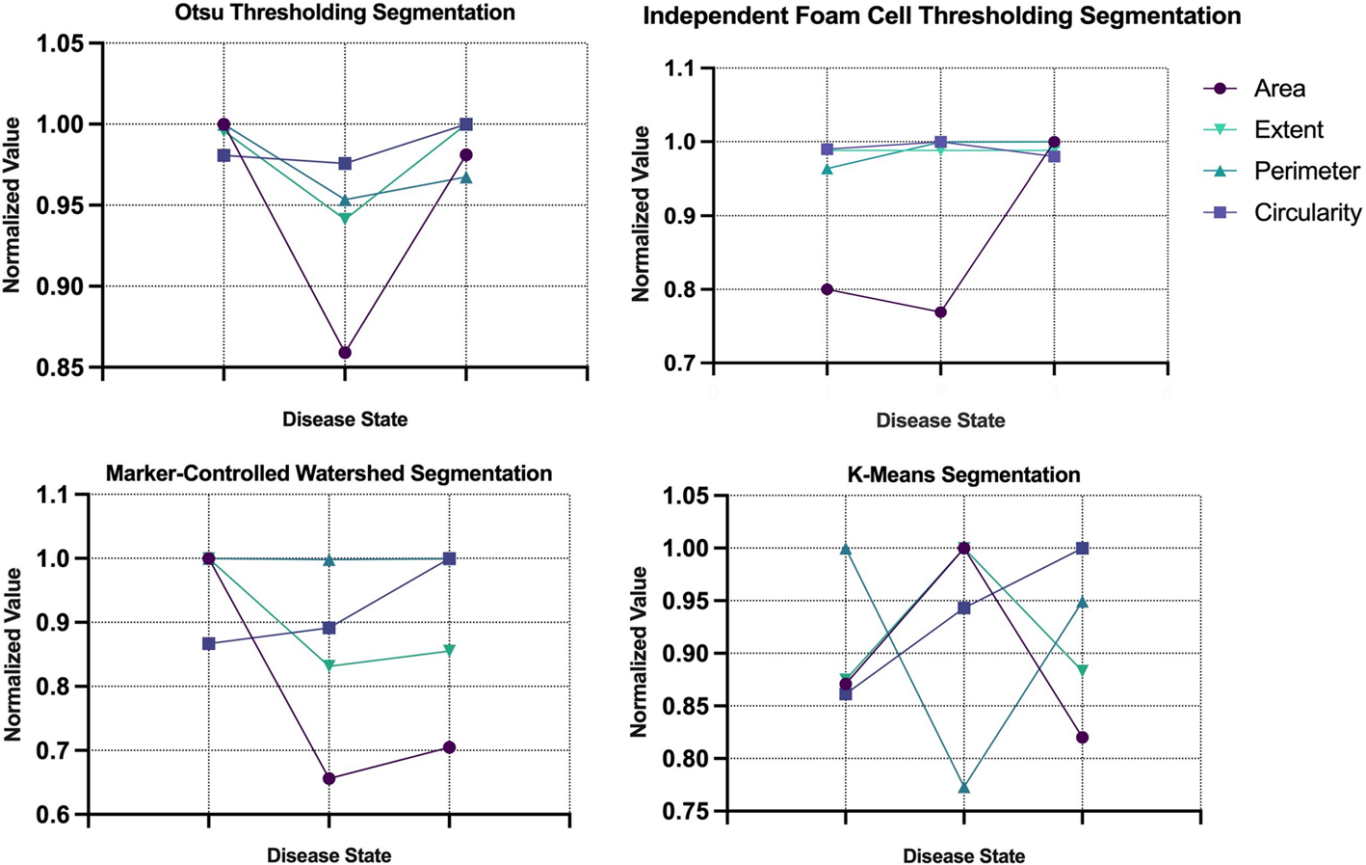
Comparison of variation in shape features for each segmentation method, OTS, IFCT, MCW and KMS and the disease states of atherosclerosis, EFS, EF and AA. The variation is normalized using the maximum of each feature. FOS, first order statistic; OTS, otsu thresholding; IFCT, independent foam cell thresholding; MCW, marker-controlled watershed; KMS, k-means segmentation; EFS, early fatty streak development; EF, early fibroatheroma; AA, advancing atheroma

**Fig. 7:**
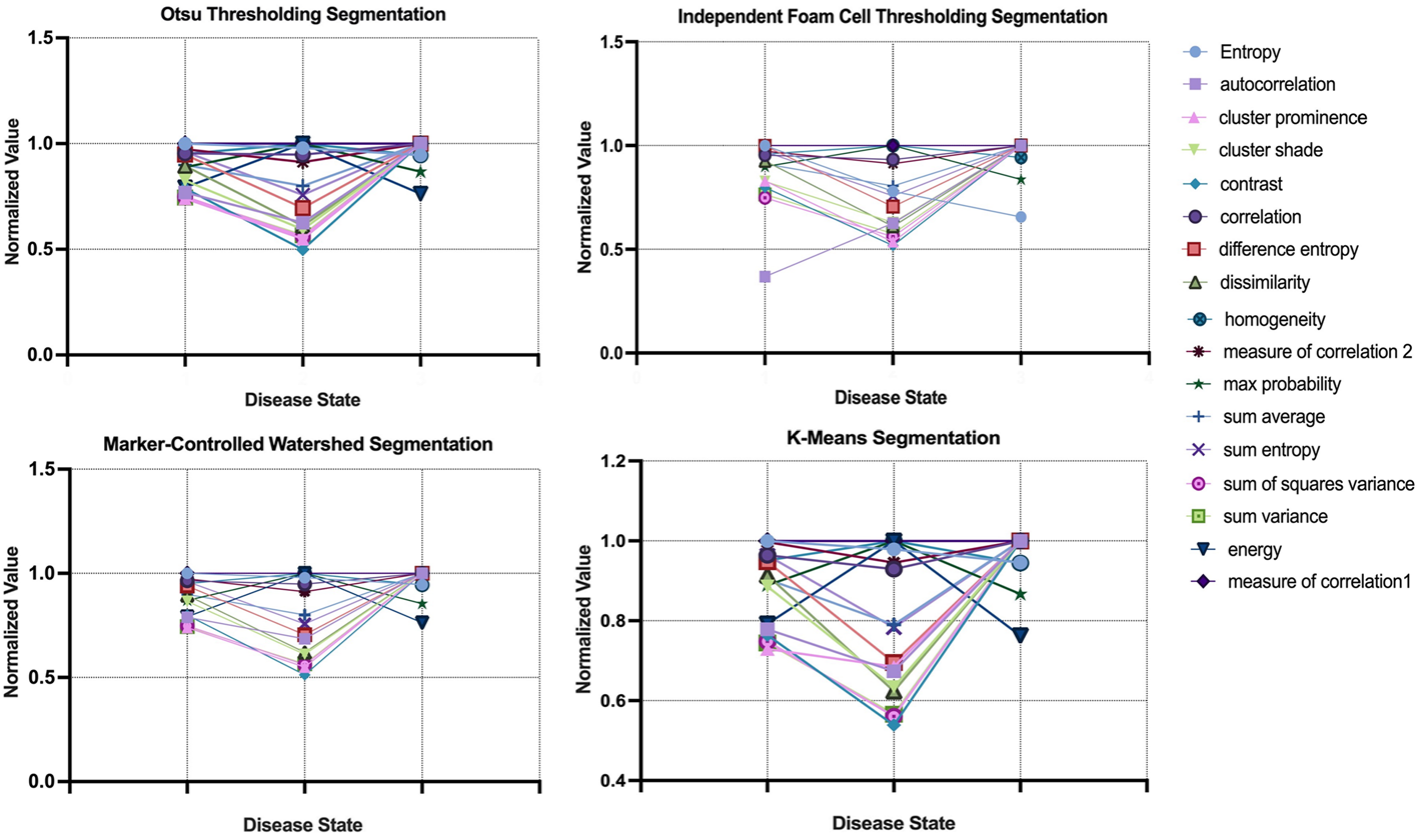
Comparison of variation in textural features for each segmentation method, OTS, IFCT, MCW and KMS and the disease states of atherosclerosis, EFS, EF and AA. The variation is normalized using the maximum of each feature. FOS, first order statistic; OTS, otsu thresholding; IFCT, independent foam cell thresholding; MCW, marker-controlled watershed; KMS, k-means segmentation; EFS, early fatty streak development; EF, early fibroatheroma; AA, advancing atheroma

In aim of improving classifier accuracy, relevant features able to objectively capture variance between EFS, EF and AA were identified. The 27 features extracted for each image were compared between the three diseased states, identifying the variation in normalized feature values between EFS, EF and AA. The averages of all twenty-seven features belonging to each imaged foam cell cluster were calculated, providing a basis to compare feature similarities and variances between the three morphological groups as presented in the following Figs. 5, 6 and 7. The non-normalized values of the features can be found in Additional file 2: Tables S3.

At an overview, there is significant overlap in feature values between EFS, EF and AA. Interclass commonalities between features do not capture variance, making it difficult for the supervised models to classify traits unique to the morphologies of the three diseased states. Not all features showed the ability to distinguish between the various atherosclerotic lesion morphologies detected using CARS imaging. Of the six FOS parameters calculated; mean, skewness, kurtosis and integrated density performed the best at capturing greatest variation amongst the three classes. The gray-levels of CARS images display atherosclerotic plaque to have higher pixel intensities found primarily on the left extrema of image histograms (greater than 150-pixel intensity). Thus, the mean intensities observed between EFS, EF and AA increase respectively; indicative of the formation of atherosclerotic plaque as the disease progresses. As expected, the sparse macrophage formation of foam cell clusters in EFS accounts for lower mean intensity. The skewness measures the asymmetry of a distribution relative to the mean value of pixel intensities. Images belonging to EF, hosted the largest score for skewness, demonstrating highest asymmetry related to gray levels distribution amongst imaged foam cell clusters. This finding is consistent with the morphology of early fibroatheroma as plaque is formed sporadically through an excessive, disarranged accumulation of macrophages. Thus, asymmetrical gray-levels capture the presence of mature and immature foam cell cluster deposits common to EF. Kurtosis calculates the distribution of gray tones, identifying if the pixel intensities are peaked or flat relative to the mean. A larger kurtosis value is indicative of greater outliers present in the pixel intensities with a peaked distribution relative to the mean. As seen with skewness, a higher kurtosis was observed in early fibroatheroma due the sporadic presence of lipid-rich structures. In advanced plaques, the observed morphologies are more structured, with defined regions spanning large areas generating stronger CARS signals. These advanced plaques lead to a more spread-out distribution of pixel intensities, showing advancing atheroma to have the lowest kurtosis values. Integrated density measuring the sum of pixel intensities of the segmented plaque demonstrated advancing atheroma as having the highest sum of intensities, appropriately noting a greater concentration of developed plaque. Complementary to the five FOS parameters, four shape features (area, extent, perimeter and circularity) were extracted for each of the 566 images. Area captured the most variation amongst the three classes, with the calculated areas observed to fluctuate amongst the four segmentation methods. Largest area was expected to be attributed to early fatty streak development. Due to the initial formation of plaque observed in EFS, the confluent accumulation of lipids spans a larger area initially, with small streaks of fat being deposited within the arterial intima. As the disease progresses, the plaque formation becomes more concentrated, defining the area as more structured and compact. However, the disparity in areas amongst classes can be attributed to segmentation inaccuracies. To specify, IFCT was the primary outlier in documenting plaque area of EFS to be the lowest amongst the four segmentation groups. Though minimal variation was observed, AA was measured as having the highest circularity amongst the three classes. However, this does not present a valuable finding as there is no expectation for circularity to be observed during plaque formation. Though extent and perimeter provided valuable metrics in measuring plaque size and ratio of pixel regions, they failed to capture much variation amongst the three classes.

Lastly, seventeen second order GLCM parameters were calculated for each of the 566 images. Homogeneity quantifies the local similarities of pixels within a ROI and is expected to be greatest for advancing atheroma due to its uniform and concentrated accumulation of macrophages. Energy features reflect the equal probability of observing gray-levels. Dominant gray levels are not observed when all the probability density functions ${\text{P}}_{\mathrm{d,h}}$(i,j) are equal. Defined areas of plaque host a greater rate of change in the intensity of pixels, in turn equating to higher observed energies. Hence, on average, AA demonstrated the highest energy amongst the three disease states.

Correlation quantifies the dependence of gray levels between two pixels separated by a distance value. Low correlation reflects gray-levels independent from one another, observing no regular structure within the image. Whereas a higher correlation marks an increased probability of one or several patterns repeating themselves.

The morphology of advancing atheroma details the plaque to be highly structured, with defined boundaries outlining lipid deposits. Entropy measures the lack of spatial organization where a higher entropy denotes greater detail within an image. Both EFS and EF are defined by their sporadic network of lipid accumulation, accurately demonstrating high entropies.

Autocorrelation is the convolution of a function with itself, comparing all possible pixel pairs and calculating their likelihood of re-appearing to characterize spatial frequency. Amongst the three classes, high autocorrelation was noted, indicating the high spatial frequencies amongst the plaque morphologies.

Cluster prominence is also a measure of asymmetry, with increased cluster prominence indicating less image symmetry. Both cluster shade and cluster prominence characterize pixel clustering within an ROI, with both features frequently observed to be highest for advancing atheroma due to the concentrated accumulation of macrophages.

Contrast measures the intensity contrast between a pixel and its neighbour. The contrast values amongst all three classes were high, reflecting non-uniformity amongst the imaged plaques. Similar to contrast, dissimilarity measures the distance between pixel pairs. Maximum probability calculates the maximal likelihood of producing the pixels of interest.

Sum average, sum variance and sum entropy respectively measure the mean of the gray level sum distribution, the dispersion (with regard to the mean) of the gray level sum distribution and the disorder related to the gray level sum distributions [50]. All of which performed sub-optimally in accounting for the variability in plaque morphology amongst the three classes.

CV and FTF evaluated the extent of variability of the twenty-seven features collected for each group (EFS, EF and AA). The CV was calculated interclass, comparing the averaged values of each of the features between the three disease states. Features displaying a CV greater than eight percent were supplied as feature predictors. To evaluate the robustness of CV, an automated filter type feature (FTF) selection algorithm measuring feature importance was used to conduct a comparative analysis. Using both CV and FTF to select features of high predictive power, the features were refined from a total of twenty-seven to an average of eighteen features for each segmentation method. The selected refined features used to boost classification accuracy are displayed in Table 2.

**Table 2:** Overview of refined features with high predictive power selected for supervised machine learning classification using CV feature selection and FTF selection

	OTS	IFCT	KMS	MCW
	FTF selection method			
Refined features				
FOS	1. Integrated density 2. Kurtosis 3. Mean intensity	1. Skewness 2. Standard deviation 3. Mean intensity 4. Standard deviation	1. Kurtosis 2. Standard deviation 3. Mean intensity	1. Kurtosis 2. Mean intensity 3. Standard deviation
Shape	4. Extent 5. Area	5. Area 6. Circularity	4. Area 5. Circularity	4. Area
GLCM	6. Sum average 7. Difference entropy 8. Sum of squares variance 9. Sum variance 10. Sum entropy 11. Measure of correlation 1 12. Homogeneity 13. Entropy 14. Measure of correlation 2 15. Autocorrelation	7. Energy 8. Homogeneity 9. Sum variance 10. Measure of correlation 2 11. Sum of squares variance 12. Sum average 13. Max probability 14. Cluster prominence 15. Autocorrelation Cluster prominence	6. Sum entropy 7. Cluster shade 8. Sum average 9. Max probability 10. Entropy 11. Sum variance 12. Autocorrelation 13. Cluster prominence 14. Dissimilarity 15. Homogeneity	5. Extent 6. Measure of correlation 2 7. Homogeneity 8. Sum of squares variance 9. Sum entropy 10. Sum average 11. Cluster shade 12. Entropy 13. Dissimilarity 14. Autocorrelation 15. Cluster prominence
Total features selected by FTF	15	15	15	15

	CV selection method			
Refined features				
FOS	1. Mean intensity 2. Skewness 3. Kurtosis 4. Integrated density	1. Standard deviation 2. Skewness 3. Kurtosis 4. Integrated Density 5. Mean Intensity	1. Mean intensity 2. Number of objects 3. Skewness 4. Kurtosis 5. Standard deviation 6. Integrated density	1. Mean intensity 2. Number of objects 3. Skewness 4. Kurtosis 5. Standard deviation 6. Integrated density
Shape	5. Area	6. Area	7. Area 8. Circularity	7. Area 8. Extent
GLCM	6. Autocorrelation 7. Cluster prominence 8. Cluster shade 9. Contrast 10. Difference entropy 11. Dissimilarity 12. Energy 13. Sum average 14. Sum entropy 15. Sum of squares variance 16. Sum variance	7. Cluster prominence 8. Cluster shade 9. Contrast 10. Difference entropy 11. Dissimilarity 12. Energy 13. Sum average 14. Sum entropy 15. Sum of squares variance 16. Sum variance 17. Autocorrelation	9. Autocorrelation 10. Cluster prominence 11. Cluster shade 12. Contrast 13. Difference entropy 14. Dissimilarity 15. Energy 16. Sum average 17. Sum entropy 18. Sum of squares variance 19. Sum variance	9. Autocorrelation 10. Cluster prominence 11. Cluster shade 12. Contrast 13. Difference entropy 14. Dissimilarity 15. Energy 16. Sum average 17. Sum entropy 18. Sum of squares variance 19. Sum variance
Total features selected by CV	16	17	19	19

It is critical to understand why certain features led to improved classification metrics. It is observed that both CV and FTF feature selection favoured textural features over FOS and shape features. However, CV did note shape features having a higher differentiation power amongst the three classes, in comparison to FTF. Skewness, kurtosis and integrated density demonstrated high CV, differentiating between EFS, EF and AA. However, FOS were predominantly eliminated by FTF. FOS statistics computed from gray-level image histograms only references the individual values of pixels, ignoring the spatial interaction between pixels. Individual pixel values from image histograms only showcase the distribution of gray levels, failing to capture objects and patterns contained within the image. These shortcomings result in FOS being regarded as a “blunt tool” for quantifying changes and spatial distribution of gray values in images [51].

In contrast, both CV and FTF heavily favoured texture features, identifying them to capture the most variation amongst classes. Haralick’s texture features enable descriptions of plaque specific heterogeneity by using GLCM as a method of quantifying the spatial resolution of neighbouring pixels in an image.

Texture features provide greater sensitivity in recognizing changes in plaque morphology by evaluating each pixel and its neighbourhood to generate a mask that is able to capture complex structures as a whole. Furthermore, shape features were also predominantly dismissed as they were shown to have minimal predictive power in capturing morphological variation amongst EFS, EF and AA, and provide minimal quantitative relevance.

The refined features identified using both CV and FTF were supplied into the three supervised classifiers. By using the refined features, it was possible to achieve significant improvement in classification accuracy and class specific recall, precision and F1 scores as reflected in Table 3.

**Table 3:**
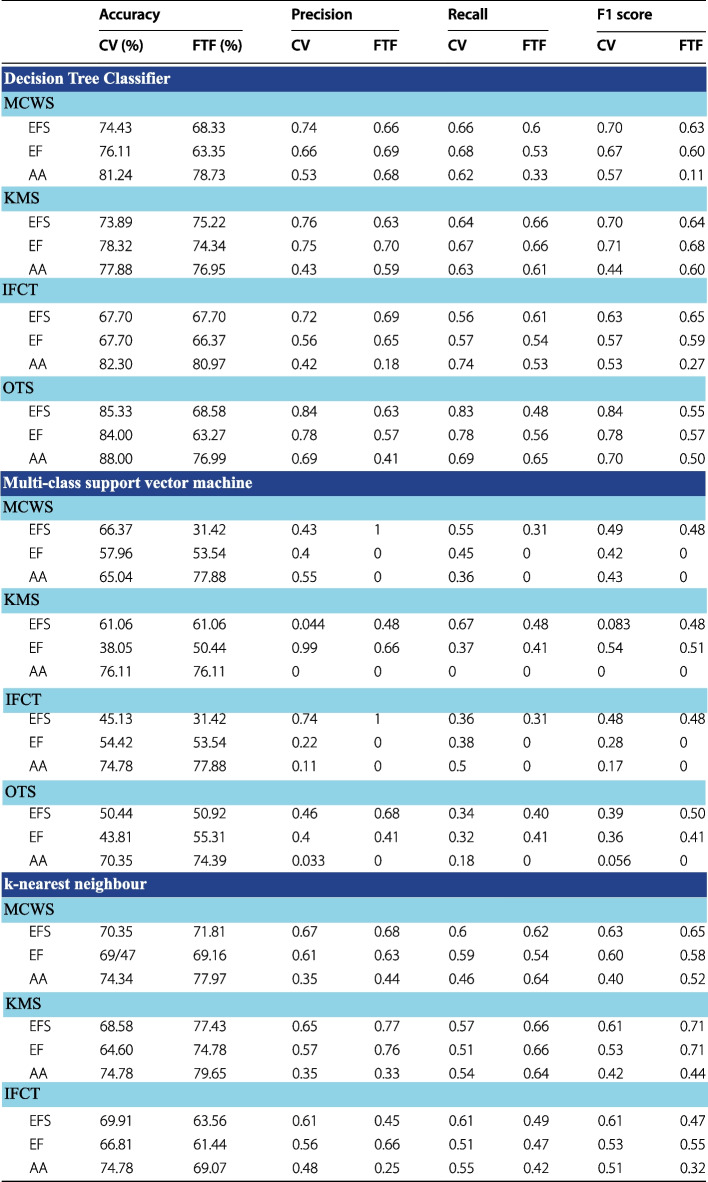

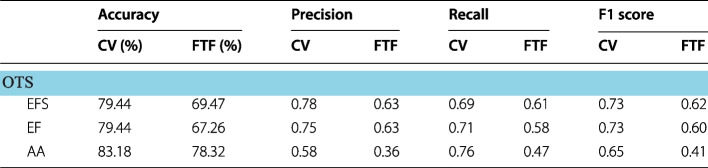
Improved performance metrics of the three supervised machine learning classifiers (MCSVM, DT and KNN) using CV and FTF refined features

EFS, early fatty streak development; EF, early fibroatheroma; AA, advancing atheroma; CV, coefficient of variation; FTF, filter type feature selection; MCWS, marker controlled watershed segmentation; IFCT, independent foam cell thresholding; OTS, Otsu thresholding segmentation

### Comparison of classifier accuracies

Supplementing refined features selected by CV and FTF, as expected, successfully improved classification metrics. Between the two feature selection methods, the features selected by CV culminated higher performance metrics amongst the three classifiers and their segmentation methods. Using CV refined features, all four segmentation methods classified using decision trees outperformed all other classifiers, trumping KNN and MCSVM. Specifically, the decision tree classifier trained using CV refined features of OTS segmented plaque performed to the highest degree of accuracy (accuracies greater than 85% as shown in Table 3) even in comparison to FTF classifiers.

Though decision trees are a more powerful classifier in comparison to KNN, preliminary results had demonstrated KNN performing with the highest classification accuracy. A major limitation within KNN’s framework is its inability to perform well using high dimensional data. Though performance metrics amongst the three classifiers did improve, MCSVM continued to perform poorly in comparison to the three other segmentation methods.

A correlation was observed amongst segmentation accuracy and classification accuracy. Refined features supplied from segmentation methods with higher Dice indices (OTS, MCW and KMS) achieved better classification performance metrics. Comparing the performances in refined features between CV and FTF, the classifiers using CV refined features on average significantly outperformed FTF features. Though FTF is an automated method to rank features, the definition of the threshold points for rankings to select only the required features and exclude noise remains unclear.

Features appearing irrelevant are removed using an arbitrary threshold. FTF ignores feature dependencies, instead evaluating the likelihood of correlation or association between features using their frequency distributions.

Furthermore, CV showed a greater disparity in the features selected, using FOS and shape features in addition to textural features. In comparison. FTF favoured textural features to draw conclusions on plaque morphology. Though textural features may provide greater sensitivity to defining patterned regions, when coupled with FOS and shape features, the morphology of the plaque is captured more cohesively and demonstrates a greater ability to differentiate between EFS, EF and AA.

## Discussion

A large overlap in selecting features with high predictive power amongst the two feature refining methods, FTF and CV was observed. Both methods placed importance on the application of textural features to capture variance in plaque morphology. This expresses the relevance of using features sensitive to recognizing patterns, spatial orientations and structural changes within a ROI. Though FOS and shape features managed to capture variation amongst plaque morphologies belonging to EFS, EF and AA, they were ranked significantly lower in importance when compared to textural features. When independently applied, shape and FOS features do not provide sufficient information. However, CV did prove that in combination with textural features, FOS features and shape features provide greater relevance in capturing detailed plaque morphologies.

Using refined features, our results showed significant improvement in classification accuracy. Based on the accuracies provided in Table 3, it is made clear that advancing atheroma and early fatty streak development (with the exception of MCSVM) can be classified to a higher degree of accuracy in comparison to early fibroatheroma. The characteristics of EFS and AA contain elements of plaque morphology unique to themselves, whereas early fibroatheroma is a combination of characteristics common to both EFS and AA. The shared characteristics of EF translated into the observed poor performance metrics of supervised classification models.

Though the models were supplied with features of high predictive power, were cross-validated and hyperparameter optimized, the dataset of EF created a gray-area amongst the three classes. The overlap in plaque morphologies contained in EF made it difficult to classify structures as being unique to EF.

In future work, to overcome this limitation, the study can be expanded to include the recognition of specific complex lesions underlying advancing atheroma. Attributes unique to advancing atheroma and its complex lesions (type V and type VI) can be leveraged to create a greater distinction in the contained morphologies of EF and AA. The formation of complex lesions are highly abstract in nature and deviate heavily from the simple accumulation of lipids observed in EFS and EF. Between Type V and Type VI atheroma, lesions are thickened by reparative collagenous tissue layers and reflect hematoma and thrombotic defects [52]. The unique characteristics of complex lesion plaque morphologies would have divided the overlapping characteristics, aiding the classifiers in distinguishing between EF and AA specifically.

To increase the level of detail of plaque morphology and identify complex lesions, CARS microscopy can be coupled with second-harmonic generation (SHG) microscopy. Specific to complex lesions, the presence of reparative collagenous fibers can be imaged using SHG. It is a viable tool for the direct visualization of extracellular collagen without the requirement of invasive tissue staining as demonstrated by Roth et al. [53]. In unison, CARS and SHG can provide greater insight into the histological changes occurring during athersosclerotic plaque progression. Through the inclusion of collagen morphology, the training dataset would be expanded to include complex lesion development of advancing atheroma [54]. Provided that the aim of the work was to develop a pipeline able to classify the onset of early-stage atherosclerosis, the features extracted provide a good foundation to differentiate amongst plaque morphologies belonging to early fatty streak development and advancing atheroma.

To improve performance metrics, it is important to acknowledge the classification accuracies being impacted by the use of a smaller image dataset. Amongst the three classes, an average of only 170 images were used to train the classifiers on distinct morphologies unique to EFS, EF and AA. With the observed implications of morphology overlap observed in EF, classifiers lacked sufficient data to guide more accurate classifications. Future research will be undertaken to expand the total dataset, by coupling CARS microscopy with SHG microscopy. This would also further detail the role of collagen in plaque morphology, providing sufficient information to differentiate amongst the complex lesions of advancing atheroma. A larger dataset would also lead to the exploration of applying deep learning, specifically the use of convolutional neural networks (CNN) to automate feature extraction and morphology classification. Lastly, the application of wrapper methods and embedded methods will be explored, testing if the generalization of the pipeline is maintained when using advanced feature refinement techniques.

## Conclusions

Although the work presented operates on a limited sample size, it shows promise in CARS ability to provide high resolution images of lipid-rich structures to track the morphological progression of atherosclerotic plaque. Through the combination of image segmentation, feature extraction and supervised machine learning algorithms, the stage to which atherosclerosis has progressed can be classified. By using supervised learning, we were able to supply features capturing the most variation amongst the three atherosclerotic lesions classes, providing justifications as to why specific features were used and their relevance to capturing plaque morphology. Manual feature selection and feature refinement provides users with an increased level of confidence when diagnosing medical conditions. The work presented provides a basis for recognizing changes in the progression of atherosclerotic plaque progression in an automated manner, while providing insight into the metrics and features used to draw potential fundamental diagnostic-relevant conclusions. These findings can be used to inform future studies focused on basic mechanisms associated with atherosclerosis progression and guide the assessment of potential novel therapeutic interventions.

## Supplementary Information

**Additional file 1: Table S1**. Overview of automated segmentation methods applied to isolate lipid-rich structures on CARS imaged atherosclerotic plaques. **Table S2**. Initial class accuracies, class precision, class recall and class F1 scores achieved using all twenty-seven extracted features.

**Additional file 2: Table S3**. Overview of the non-normalized descriptive statistics of FOS features, shape features and texture features.

## Data Availability

The datasets used and/or analysed during the current study available from the corresponding author on reasonable request.

## Abbreviations

CARS: Coherent anti stokes Raman spectroscopy
EFS: Early fatty streak development
EF: Early fibroatheroma
AA: Advancing atheroma
CV: Coefficient of variation
FTF: Filter type feature selection
MCWS: Marker controlled watershed segmentation
IFCT: Independent foam cell thresholding
KMS: K-means segmentation
OTS: Otsu thresholding segmentation
MCSVM: Multi-class support vector machine
kNN: K-nearest neighbour
DT: Decision tree
GLCM: Gray level co-occurence matrix
FOS: First order statistics
SHG: Second harmonic generation
WHHLMI: Watanabe heritable hyperlipidemic model
PET: Positron emission tomography
IVUS: Intravascular ultrasound
MRI: Magnetic resonance imaging
OCT: Optical computed tomography
