# Supplementary material for: Development of an image classification pipeline for atherosclerotic plaques assessment using supervised machine learning
Source: BMC Bioinformatics. 2022 Dec 14;23:542. doi: 10.1186/s12859-022-05059-1 (PMC9753267; doi:10.1186/s12859-022-05059-1)
Supplement: Supplementary file 1 — Additional file 1: Table S1. Overview of automated segmentation methods applied to isolate lipid-rich structures on CARS imaged atherosclerotic plaques. Table S2. Initial class accuracies, class precision, class recall and class F1 scores achieved using all twenty-seven extracted features. [file 12859_2022_5059_MOESM1_ESM.pdf]

Development of an Image Classification Pipeline for Atherosclerotic Plaques Assessment using Supervised Machine Learning

Natasha N. Kunchur[1], Leila B. Mostaço-Guidolin[1]

SUPPLEMENTARY MATERIAL

◊ Details about plaque segmentation methods

Supplemental Table 1 Overview of automated segmentation methods applied to isolate lipid-rich structures on CARS imaged atherosclerotic plaques.

Segmentation Method	Application
**Otsu Thresholding (OTS)**	A classical segmentation method computing a global threshold T from a grayscale image. Otsu's method selects a threshold that minimizes the intraclass variance of the thresholded black and white pixels from histogram counts. The optimal threshold value is computed by minimizing the sum of the weighted group variances; the weights are the probability of each respective group.<br>Otsu's thresholding corresponds to the linear discriminant criteria under the assumption that the image consists of only a foreground and background. The artifacts contained within the background are ignored. Otsu is set to minimize the overlap of class distributions [1].
**Independent Foam Cell Thresholding (IFCT)**	Novel segmentation method built on the foundation of Otsu Thresholding, where each identified foam cell cluster within the plaque is segmented independently from its respective backgrounds. In contrast to Otsu Thresholding, the image is not directly split into a foreground and background, marking the entirety of the imaged plaque as the foreground. Rather, each identified foam cell cluster is treated as its own entity, where the optimal threshold value is computed by minimizing the sum of the weighted group variances between the foam cell cluster and its respective surrounding areas (which is inclusive of plaque).
**Marker Controlled Watershed (MCW)**	Marker Controlled Watershed segmentation is a region-based segmentation method utilizing image morphology, often observed to be more sensitive to detecting weaker edges. The watershed transform regards an image as a topographic landscape containing ridges and valleys, finding "catchment basins" and "watershed ridge lines" where light pixels are marked as high and dark pixels marked as low. MCW defines the elevation values of the landscape by the gradient magnitude of the image. The watershed transform decomposes an image into catchment basins. Using MCW, image locations belonging to a ROI, the plaque, can be specifically targeted [2]. Marker-controlled Watershed is expected to be more sensitive to capturing textural variations and details during segmentation.
**K-Means (KMS)**	An unsupervised clustering algorithm used to classify the image into distinct groupings using a defined number of k-clusters to partition the image. Using K-means, each image was segmented into two k-regions ($k=2$), defining the

	foreground as the plaque and background as noise. To improve the accuracy of k-means segmentation, textural and spatial information for each pixel contained within the image was supplemented as extra data into the k-means algorithm. Textures of each pixel were defined using a set of twenty-four Gabor filters, covering six wavelengths of the sinusoidal carrier, specified as equal to 2, in pixels/cycle. Wavelength values typically range from 2 up to the hypotenuse length of the input image. In addition to wavelengths, four orientations of the filter in degrees, specified as at 90,180, 270 and 360 degrees. Formally, the orientation is the normal direction to the sinusoidal plane wave.

◊ Initial classifier performance metrics (prior to feature refinement)

Supplemental Table 2 Initial class accuracies, class precision, class recall and class F1 scores achieved using all twenty-seven extracted features

Decision Tree Classifier	**MCWS**				
		Accuracy	*Precision*	*Recall*	*F1 Score*
	**EFS**	64.16%	0.66	0.52	0.58
	**EF**	65.93%	0.49	0.51	0.5
	**AA**	77.88%	0.44	0.64	0.52
	**K-Means Segmentation**				
		Accuracy	*Precision*	*Recall*	*F1 Score*
	**EFS**	61.50%	0.58	0.44	0.5
	**EF**	61.95%	0.43	0.58	0.49
	**AA**	74.78%	0.47	0.46	0.47
	**IFCT**				
		Accuracy	*Precision*	*Recall*	*F1 Score*
	**EFS**	61.50%	0	0	0
	**EF**	41.15%	1	0.41	0.58
	**AA**	79.65%	0	0	0
	**OTS**				
		Accuracy	*Precision*	*Recall*	*F1 Score*
	**EFS**	69.47%	0.66	0.64	0.65
	**EF**	66.37%	0.68	0.54	0.6
	**AA**	83.63%	0.3	0.68	0.41
Multi-class Support Vector Machine	**MCWS**				
		Accuracy	*Precision*	*Recall*	*F1 Score*
	**EFS**	61.50%	0.23	0.57	0.33
	**EF**	63.72%	0.037	0.43	0.068
	**AA**	37.61%	0.89	0.26	0.4
	**K-Means Segmentation**				
		Accuracy	*Precision*	*Recall*	*F1 Score*
	**EFS**	38.94%	0.94	0.39	0.55
	**EF**	63.72%	0	0	0
	**AA**	74.78%	0.056	0.33	0.095
	**IFCT**				
		Accuracy	*Precision*	*Recall*	*F1 Score*
	**EFS**	39.82%	0	0.4	0.57
	**EF**	62.83%	0	0	0
	**AA**	76.99%	1	0	0
	**OTS**				
		Accuracy	*Precision*	*Recall*	*F1 Score*
	**EFS**	47.79%	0.78	0.14	0.51

	**EF**	61.06%	0.11	0.48	0.19
	**AA**	60.86%	0.1	0.38	0.12
**k-Nearest Neighbour**	**MCWS**				
		Accuracy	*Precision*	*Recall*	*F1 Score*
	**EFS**	7.70%	0.16%	0.66%	0.72%
	**EF**	70.05%	0.59%	0.58%	0.59%
	**AA**	74.19%	0.16%	0.28%	0.2%
	**K-Means Segmentation**				
		Accuracy	*Precision*	*Recall*	*F1 Score*
	**EFS**	65.04%	0.47	0.6	0.53
	**EF**	57.52%	0.56	0.42	0.48
	**AA**	72.12%	0.35	0.4	0.38
	**IFCT**				
		Accuracy	*Precision*	*Recall*	*F1 Score*
	**EFS**	62.28%	0.41	0.51	0.46
	**EF**	59.65%	0.62	0.51	0.56
	**AA**	71.19%	0.41	0.43	0.42
	**OTS**				
		Accuracy	*Precision*	*Recall*	*F1 Score*
	**EFS**	70.35%	0.68	0.63	0.65
	**EF**	70.80%	0.63	0.63	0.63
	**AA**	80.97%	0.45	0.55	0.49

EFS - early fatty streak development, EF - early fibroatheroma, AA - advancing atheroma, CV- coefficient of variation, FTF – filter type feature selection, MCWS- marker controlled watershed segmentation, IFCT – independent foam cell thresholding, OTS – Otsu thresholding segmentation (OTS).

References

1. Yousefi, J.: Image binarization using otsu thresholding algorithm. Ontario, Canada: University of Guelph (2011)
2. Preim, B., Botha, C.P.: Visual Computing for Medicine: Theory, Algorithms, and Applications. Newnes, (2013)
