# Supplementary material for: Development of an image classification pipeline for atherosclerotic plaques assessment using supervised machine learning
Source: BMC Bioinformatics. 2022 Dec 14;23:542. doi: 10.1186/s12859-022-05059-1 (PMC9753267; doi:10.1186/s12859-022-05059-1)
Supplement: Supplementary file 2 — Additional file 2: Table S3. Overview of the non-normalized descriptive statistics of FOS features, shape features and texture features. [file 12859_2022_5059_MOESM2_ESM.pdf]

Development of an Image Classification Pipeline for Atherosclerotic Plaques Assessment using Supervised Machine Learning

Natasha N. Kunchur1, Leila B. Mostaço-Guidolin1

SUPPLEMENTARY MATERIAL

à **Descriptive statistics of FOS features, shape features and texture features**

Supplemental Table 3 Overview of the non-normalized descriptive statistics of FOS features, shape features and texture features

	area	Circularity	Perimeter	Extent	Entropy	number of objects	skewness	kurtosis	standard deviation	integrated density
**Otsu Segmentation**										
**Early Fatty Streak Development**										
Minimum	11264	0.03556	920.2	0.2522	2.929	380	0.01209	0.0001104	0.06845	562682
25% Percentile	82651	0.1193	2318	0.4812	4.697	1947	0.0575	0.0009744	0.4495	3824939
Median	111979	0.1742	2817	0.5649	5.175	2695	0.2068	0.007087	0.4819	6384786
75% Percentile	145467	0.2491	3315	0.6634	5.675	3471	0.555	0.03533	0.4959	12139616
Maximum	260910	0.7869	5922	0.9953	6.887	20853	5.128	16.7	0.5	28399145
Range	249646	0.7514	5002	0.7431	3.958	20473	5.116	16.7	0.4315	27836463
Mean	117022	0.202	2856	0.5732	5.128	3020	0.5941	0.5889	0.4576	8501043
Std. Deviation	48416	0.1154	788.4	0.1343	0.7577	2262	0.977	2.334	0.06692	6516143
Std. Error of M	3325	0.007922	54.15	0.009223	0.05204	155.3	0.0671	0.1603	0.004596	447531
**Early Fibroatheroma**										
Minimum	10851	0.03425	733	0.2492	2.579	468	0.01129	0.00009703	0.09921	324461
25% Percentile	60856	0.1077	2148	0.4243	4.627	1695	0.08509	0.001795	0.4044	1830728
Median	93587	0.1658	2660	0.5272	5.085	2481	0.8217	0.07136	0.4613	3324004
75% Percentile	130250	0.2343	3177	0.6464	5.546	3460	1.955	0.4771	0.4938	10198954
Maximum	259538	0.747	6553	0.9901	6.473	18915	6.065	15.22	0.5	28839865
Range	248687	0.7127	5820	0.7408	3.894	18447	6.053	15.22	0.4008	28515404
Mean	100528	0.1971	2723	0.5418	5.019	3164	1.191	0.9603	0.4314	6631936
Std. Deviation	54304	0.1391	867	0.1635	0.762	3529	1.345	2.538	0.08844	6750332
Std. Error of M	4691	0.01202	74.9	0.01412	0.06583	255.3	0.1162	0.2192	0.00764	583140
**Advancing Atheroma**										
Minimum	6860	0.04719	452.6	0.2621	2.804	219	0.009462	0.00007191	0.1596	530190
25% Percentile	69412	0.1296	2264	0.4713	4.434	1810	0.02968	0.0003982	0.4258	3402725
Median	108491	0.1763	2723	0.5662	4.848	2638	0.1062	0.002638	0.467	7103563
75% Percentile	159963	0.2498	3222	0.6899	5.214	3342	0.5129	0.0378	0.4914	16711137
Maximum	240973	0.4981	5011	0.9192	6.682	11965	4.129	12.29	0.5	33716323
Range	234113	0.4509	4558	0.6571	3.878	11746	4.12	12.29	0.3404	33186133
Mean	114808	0.1981	2763	0.5756	4.849	2823	0.4753	0.2141	0.4475	10389534
Std. Deviation	54558	0.09856	712.8	0.1439	0.6449	1549	0.7714	1.02	0.05879	8319920
Std. Error of M	3695	0.006675	48.28	0.009744	0.04368	104.9	0.05224	0.06907	0.003982	563496
**Independent Foam Cell Thresholding Segmentation**										
**Early Fatty Streak Development**										
Minimum	7070	0.00352	0.6986	5.367	2.929	138	0.01209	0.0001104	10.79	74942
25% Percentile	12786	0.1371	0.7525	11.42	4.697	547.8	0.0575	0.0009744	63.41	1123639
Median	15910	0.1856	0.7717	13.47	5.175	807	0.2068	0.007087	103.3	1852859
75% Percentile	37001	0.2872	0.7975	18.18	5.675	1010	0.555	0.03533	142	4919904
Maximum	108038	0.6612	0.902	37.63	6.887	5980	5.128	16.7	1803	17697900
Range	100968	0.6589	0.2034	32.26	3.958	5842	5.116	16.7	1792	17622958
Mean	26969	0.1976	0.7774	14.97	5.128	836.9	0.5941	0.5889	120.8	3422799
Std. Deviation	22765	0.067	0.03567	4.924	0.7577	505.7	0.977	2.334	138.6	3606752
Std. Error of M	1563	0.76	0.00245	0.3382	0.05204	34.73	0.0671	0.1603	9.516	247713
**Early Fibroatheroma**										
Minimum	4687	0.00543	0.7122	2.09	2.579	156	0.01129	0.00009703	13.06	34599
25% Percentile	12078	0.1571	0.7741	11.67	4.627	434.8	0.08509	0.001795	46.1	620563
Median	17026	0.1786	0.7983	13.95	5.085	644	0.8217	0.07136	80.13	1128330
75% Percentile	32933	0.2721	0.8249	17.38	5.546	869	1.955	0.4771	118.8	2689434
Maximum	110391	0.6748	0.933	27.43	6.473	5477	6.065	15.22	1058	12446968
Range	105704	0.6538	0.2208	25.34	3.894	5321	6.053	15.22	1045	12412369
Mean	25921	0.1832	0.8063	14.87	5.019	746.4	1.191	0.9603	99.31	2314200
Std. Deviation	21311	0.08	0.0441	4.852	0.762	607	1.345	2.538	107.8	2629765
Std. Error of M	1841	0.7142	0.003809	0.4192	0.06583	52.44	0.1162	0.2192	9.311	227177
**Advancing Atheroma**										
Minimum	5371	0.01345	0.6999	5.627	2.804	65	0.009462	0.00007191	3.251	103788
25% Percentile	11424	0.1754	0.7746	11.45	4.434	488.8	0.02968	0.0003982	52.58	998302
Median	16976	0.1652	0.7945	14.07	4.848	704	0.1062	0.002638	90.26	2019332
75% Percentile	56381	0.2235	0.8292	18.44	5.214	943.3	0.5129	0.0378	143.1	6557348
Maximum	132562	0.4641	0.9458	27.69	6.682	1947	4.129	12.29	305	16241296
Range	127191	0.6453	0.2458	22.07	3.878	1882	4.12	12.29	301.7	16137508
Mean	33698	0.4631	0.8068	15.15	4.849	739.2	0.4753	0.2141	100.7	3735419
Std. Deviation	31173	0.0652	0.04601	4.864	0.6449	329.3	0.7714	1.02	60.98	3466001
Std. Error of M	2111	0.8921	0.003117	0.3294	0.04368	22.3	0.05224	0.06907	4.13	234747
**Marker-controlled waterhsed segmentation**										
**Early Fatty Streak Development**										
Minimum	4417	0.007278	788.8	0.2325	2.929	1	0.01209	0.0001104	0	488868
25% Percentile	79170	0.01791	2003	0.4844	4.697	1362	0.0575	0.0009744	0	4197007
Median	137660	0.02785	6081	0.6003	5.175	1792	0.2068	0.007087	0.4371	7055760
75% Percentile	262144	0.8213	8751	1	5.675	2432	0.555	0.03533	0.4898	11647772
Maximum	262144	0.8213	14784	1	6.887	4693	5.128	16.7	0.5	26870443
Range	257727	0.814	13995	0.7675	3.958	4692	5.116	16.7	0.5	26381575
Mean	151590	0.2518	6042	0.6723	5.128	1915	0.5941	0.5889	0.3186	8259320
Std. Deviation	82937	0.359	3381	0.2371	0.7577	949.1	0.977	2.334	0.2089	5626243
Std. Error of M	5696	0.02466	232.2	0.01628	0.05204	65.19	0.0671	0.1603	0.01435	386412
**Early Fibroatheroma**										
Minimum	4471	0.00526	665.1	0.2548	2.579	1	0.01129	0.000097	0	183741
25% Percentile	55699	0.01731	3941	0.4191	4.627	485	0.08509	0.001795	0.3288	1535065
Median	79608	0.0254	6174	0.5208	5.085	1171	0.8217	0.07136	0.4309	3405401
75% Percentile	117222	0.04184	7910	0.6343	5.546	2133	1.955	0.4771	0.4802	9257158
Maximum	262144	0.8213	16023	1	6.473	6284	6.065	15.22	0.4997	27216667
Range	257673	0.816	15358	0.7452	3.894	6283	6.053	15.22	0.4997	27032926
Mean	99466	0.1176	6031	0.5591	5.019	1372	1.191	0.9603	0.3737	5830406
Std. Deviation	70596	0.2516	2915	0.1946	0.762	1146	1.345	2.538	0.155	5613747
Std. Error of M	6099	0.02174	251.8	0.01681	0.06583	99	0.1162	0.2192	0.01339	484954
**Advancing Atheroma**										
Minimum	3422	0.005983	342.4	0.2615	2.804	1	0.009462	0.0000719	0	250222
25% Percentile	53542	0.01614	2950	0.4266	4.434	1214	0.02968	0.0003982	0.2864	2802237
Median	86355	0.02347	6355	0.5227	4.848	1948	0.1062	0.002638	0.4307	6784894
75% Percentile	135252	0.04393	8273	0.634	5.214	2752	0.5129	0.0378	0.4869	11637826
Maximum	262144	0.8213	12173	1	6.682	6968	4.129	12.29	0.5	27960057
Range	258722	0.8153	11831	0.7385	3.878	6967	4.12	12.29	0.5	27709835
Mean	106845	0.1489	6037	0.5749	4.849	2034	0.4753	0.2141	0.3556	8114792
Std. Deviation	76931	0.2864	3020	0.2082	0.6449	1162	0.7714	1.02	0.1734	6064571
Std. Error of M	5210	0.0194	204.5	0.0141	0.04368	78.69	0.05224	0.06907	0.01174	410745
**K-means segmentation**										
**Early Fatty Streak Development**										
Minimum	326	0.003777	227.3	0.1547	2.929	1	0.01209	0.0001104	0.03524	14489
25% Percentile	35791	0.0141	3167	0.3502	4.697	1	0.0575	0.0009744	0.3451	1802180
Median	76459	0.02103	6027	0.431	5.175	1	0.2068	0.007087	0.4548	5031296
75% Percentile	94873	0.03704	7942	0.521	5.675	1	0.555	0.03533	0.481	9771564
Maximum	147201	0.5208	14353	0.7903	6.887	1	5.128	16.7	0.5	27266347
Range	146875	0.517	14126	0.6356	3.958	0	5.116	16.7	0.4648	27251858
Mean	67498	0.04021	5759	0.4345	5.128	1	0.5941	0.5889	0.3856	6699202
Std. Deviation	39940	0.06458	3018	0.1302	0.7577	0	0.977	2.334	0.1418	6385527
Std. Error of M	2743	0.004436	207.3	0.008942	0.05204	0	0.0671	0.1603	0.009738	438560
**Early Fibroatheroma**										
Minimum	334	0.006437	351.6	0.1568	2.579	1	0.01129	0.000097	0.03567	10521
25% Percentile	48859	0.02056	2629	0.3921	4.627	1	0.08509	0.001795	0.3894	1312047
Median	87711	0.03846	3975	0.501	5.085	1	0.8217	0.07136	0.4693	3137282
75% Percentile	110966	0.1217	6287	0.6068	5.546	1	1.955	0.4771	0.4939	6170677
Maximum	203827	0.5879	10822	0.8226	6.473	1	6.065	15.22	0.5	25500502
Range	203493	0.5815	10470	0.6657	3.894	0	6.053	15.22	0.4643	25489981

	autocorrelation	cluster prominence	cluster shade	contrast	correlation	difference entropy	dissimilarity	energy	measure of correlation1	measure of correlation2	max probability	sum average	sum entropy	sum of squares variance	sum variance	homogeneity
**Otsu Segmentation**																
**Early Fatty Streak Development**																
Minimum	1	0.0002409	0.0001645	0.000088	-0.0001554	0.0009097	0.000088	0.1581	-2	0.04815	0.3636	2	0.00103	0.00005356	0.0001262	0.705
25% Percentile	2.308	61.79	9.451	0.2747	0.7032	0.5609	0.2168	0.2752	-2	0.975	0.5064	2.726	1.065	0.6515	2.361	0.7905
Median	4.23	287.3	31.57	0.6642	0.7746	0.8288	0.3998	0.3731	-1.999	0.9941	0.5996	3.438	1.511	1.625	5.733	0.8429
75% Percentile	8.778	997.8	82.92	1.492	0.8447	1.049	0.6063	0.5235	-1.999	0.998	0.7124	4.633	1.846	4.414	16.34	0.8997
Maximum	24.39	2166	170.1	3.88	0.9576	1.388	1.076	0.9998	-1.983	0.9995	0.9999	7.96	2.26	9.953	37.44	1
Range	23.39	2166	170.1	3.88	0.9578	1.387	1.076	0.8417	0.01686	0.9514	0.6363	5.96	2.259	9.953	37.44	0.295
Mean	6.306	558.1	47.96	0.9456	0.747	0.7771	0.4173	0.4315	-1.999	0.931	0.6246	3.808	1.396	2.656	9.677	0.8498
Std. Deviation	5.34	602.4	45.73	0.8572	0.1571	0.3446	0.259	0.2195	0.001752	0.1926	0.1649	1.415	0.5855	2.589	9.588	0.07591
Std. Error of M	0.3668	41.38	3.14	0.05887	0.01079	0.02367	0.01779	0.01508	0.0001203	0.01323	0.01133	0.09717	0.04021	0.1778	0.6585	0.005214
**Early Fibroatheroma**																
Minimum	1.001	0.006331	0.00294	0.0003271	0.2087	0.002951	0.0003271	0.152	-2	0.1153	0.3694	2.001	0.005368	0.0004589	0.001294	0.6911
25% Percentile	1.316	2.744	0.9079	0.06566	0.6674	0.2359	0.06242	0.2953	-2	0.8696	0.5219	2.22	0.516	0.1373	0.4586	0.8035
Median	2.252	40.08	5.394	0.1751	0.7769	0.4559	0.163	0.5326	-1.999	0.974	0.7157	2.773	1.027	0.4887	1.754	0.9207
75% Percentile	7.694	737.5	64.9	1.004	0.8578	0.9588	0.5064	0.7796	-1.999	0.9976	0.881	4.329	1.733	3.593	12.92	0.9692
Maximum	24.68	2102	177.3	3.695	0.9422	1.344	0.9562	0.9988	-1.993	0.9995	0.9994	8.034	2.256	10.23	37.54	0.9998
Range	23.68	2102	177.3	3.694	0.7335	1.341	0.9559	0.8468	0.006565	0.8842	0.6301	6.033	2.25	10.23	37.54	0.3088
Mean	5.133	414.5	34.49	0.5985	0.7422	0.5702	0.2849	0.5447	-1.999	0.8728	0.7028	3.388	1.1	1.99	7.359	0.8933
Std. Deviation	5.578	606.5	46.57	0.7716	0.1456	0.3857	0.2672	0.2651	0.0009816	0.2194	0.1931	1.52	0.6922	2.667	9.972	0.08426
Std. Error of M	0.4819	52.4	4.023	0.06666	0.01258	0.03332	0.02308	0.0229	0.0000848	0.01896	0.01668	0.1313	0.0598	0.2304	0.8615	0.007279
**Advancing Atheroma**																
Minimum	1.001	0.001845	-5.726	0.0003482	0.2696	0.003119	0.0003482	0.17	-2	0.09917	0.326	2.001	0.004081	0.0002677	0.0007228	0.6983
25% Percentile	2.339	73.39	8.861	0.2838	0.7486	0.5757	0.2226	0.2502	-1.999	0.9782	0.481	2.74	1.113	0.6324	2.22	0.7818
Median	5.559	611.3	57.45	0.8049	0.8121	0.8772	0.4392	0.3506	-1.999	0.996	0.5808	3.703	1.61	2.547	9.448	0.8325
75% Percentile	13.68	1483	99.14	1.958	0.8531	1.121	0.7086	0.4966	-1.999	0.9986	0.695	5.532	1.939	6.64	24.53	0.897
Maximum	30.43	2104	175.3	4.619	0.9304	1.405	1.174	0.9991	-1.99	0.9995	0.9996	9.083	2.223	10.96	41.52	0.9998
Range	29.43	2104	181	4.618	0.6609	1.402	1.174	0.8291	0.009584	0.9003	0.6736	7.082	2.219	10.96	41.52	0.3015
Mean	8.204	754	58.08	1.2	0.7828	0.82	0.4667	0.4154	-1.999	0.9566	0.6092	4.235	1.453	3.55	13	0.8436
Std. Deviation	6.979	692	48.85	1.079	0.1113	0.3536	0.2888	0.2087	0.0008453	0.1121	0.1627	1.761	0.5698	3.239	11.97	0.07618
Std. Error of M	0.4727	46.87	3.309	0.07307	0.007538	0.02395	0.01956	0.01413	0.00005725	0.007591	0.01102	0.1193	0.03859	0.2194	0.8107	0.00516
**Independent Foam Cell Thresholding Segmentation**																
**Early Fatty Streak Development**																
Minimum	1	0.0002409	0.0001645	0.000088	-0.0001554	0.0009097	0.000088	0.1581	-2	0.04815	0.3636	2	0.00103	0.00005356	0.0001262	0.705
25% Percentile	2.308	61.79	9.451	0.2747	0.7032	0.5609	0.2168	0.2752	-2	0.975	0.5064	2.726	1.065	0.6515	2.361	0.7905
Median	4.23	287.3	31.57	0.6642	0.7746	0.8288	0.3998	0.3731	-1.999	0.9941	0.5996	3.438	1.511	1.625	5.733	0.8429
75% Percentile	8.778	997.8	82.92	1.492	0.8447	1.049	0.6063	0.5235	-1.999	0.998	0.7124	4.633	1.846	4.414	16.34	0.8997
Maximum	24.39	2166	170.1	3.88	0.9576	1.388	1.076	0.9998	-1.983	0.9995	0.9999	7.96	2.26	9.953	37.44	1
Range	23.39	2166	170.1	3.88	0.9578	1.387	1.076	0.8417	0.01686	0.9514	0.6363	5.96	2.259	9.953	37.44	0.295
Mean	6.306	558.1	47.96	0.9456	0.747	0.7771	0.4173	0.4315	-1.999	0.931	0.6246	3.808	1.396	2.656	9.677	0.8498
Std. Deviation	5.34	602.4	45.73	0.8572	0.1571	0.3446	0.259	0.2195	0.001752	0.1926	0.1649	1.415	0.5855	2.589	9.588	0.07591
Std. Error of M	0.3668	41.38	3.14	0.05887	0.01079	0.02367	0.01779	0.01508	0.0001203	0.01323	0.01133	0.09717	0.04021	0.1778	0.6585	0.005214
**Early Fibroatheroma**																
Minimum	1.001	0.006331	0.00294	0.0003271	0.2087	0.002951	0.0003271	0.152	-2	0.1153	0.3694	2.001	0.005368	0.0004589	0.001294	0.6911
25% Percentile	1.316	2.744	0.9079	0.06566	0.6674	0.2359	0.06242	0.2953	-2	0.8696	0.5219	2.22	0.516	0.1373	0.4586	0.8035
Median	2.252	40.08	5.394	0.1751	0.7769	0.4559	0.163	0.5326	-1.999	0.974	0.7157	2.773	1.027	0.4887	1.754	0.9207
75% Percentile	7.694	737.5	64.9	1.004	0.8578	0.9588	0.5064	0.7796	-1.999	0.9976	0.881	4.329	1.733	3.593	12.92	0.9692
Maximum	24.68	2102	177.3	3.695	0.9422	1.344	0.9562	0.9988	-1.993	0.9995	0.9994	8.034	2.256	10.23	37.54	0.9998
Range	23.68	2102	177.3	3.694	0.7335	1.341	0.9559	0.8468	0.006565	0.8842	0.6301	6.033	2.25	10.23	37.54	0.3088
Mean	5.133	414.5	34.49	0.5985	0.7422	0.5702	0.2849	0.5447	-1.999	0.8728	0.7028	3.388	1.1	1.99	7.359	0.8933
Std. Deviation	5.578	606.5	46.57	0.7716	0.1456	0.3857	0.2672	0.2651	0.0009816	0.2194	0.1931	1.52	0.6922	2.667	9.972	0.08426
Std. Error of M	0.4819	52.4	4.023	0.06666	0.01258	0.03332	0.02308	0.0229	0.0000848	0.01896	0.01668	0.1313	0.0598	0.2304	0.8615	0.007279
**Advancing Atheroma**																
Minimum	1.001	0.001845	-5.726	0.0003482	0.2696	0.003119	0.0003482	0.17	-2	0.09917	0.326	2.001	0.004081	0.0002677	0.0007228	0.6983
25% Percentile	2.339	73.39	8.861	0.2838	0.7486	0.5757	0.2226	0.2502	-1.999	0.9782	0.481	2.74	1.113	0.6324	2.22	0.7818
Median	5.559	611.3	57.45	0.8049	0.8121	0.8772	0.4392	0.3506	-1.999	0.996	0.5808	3.703	1.61	2.547	9.448	0.8325
75% Percentile	13.68	1483	99.14	1.958	0.8531	1.121	0.7086	0.4966	-1.999	0.9986	0.695	5.532	1.939	6.64	24.53	0.897
Maximum	30.43	2104	175.3	4.619	0.9304	1.405	1.174	0.9991	-1.99	0.9995	0.9996	9.083	2.223	10.96	41.52	0.9998
Range	29.43	2104	181	4.618	0.6609	1.402	1.174	0.8291	0.009584	0.9003	0.6736	7.082	2.219	10.96	41.52	0.3015
Mean	8.204	754	58.08	1.2	0.7828	0.82	0.4667	0.4154	-1.999	0.9566	0.6092	4.235	1.453	3.55	13	0.8436
Std. Deviation	6.979	692	48.85	1.079	0.1113	0.3536	0.2888	0.2087	0.0008453	0.1121	0.1627	1.761	0.5698	3.239	11.97	0.07618
Std. Error of M	0.4727	46.87	3.309	0.07307	0.007538	0.02395	0.01956	0.01413	0.00005725	0.007591	0.01102	0.1193	0.03859	0.2194	0.8107	0.00516
**Marker-controlled waterhsed segmentation**																
**Early Fatty Streak Development**																
Minimum	1	0.0002409	0.0001645	0.000088	-0.0001554	0.0009097	0.000088	0.1581	-2	0.04815	0.3636	2	0.00103	0.0000536	0.0001262	0.705
25% Percentile	2.308	61.79	9.451	0.2747	0.7032	0.5609	0.2168	0.2752	-2	0.975	0.5064	2.726	1.065	0.6515	2.361	0.7905
Median	4.23	287.3	31.57	0.6642	0.7746	0.8288	0.3998	0.3731	-1.999	0.9941	0.5996	3.438	1.511	1.625	5.733	0.8429
75% Percentile	8.778	997.8	82.92	1.492	0.8447	1.049	0.6063	0.5235	-1.999	0.998	0.7124	4.633	1.846	4.414	16.34	0.8997
Maximum	24.39	2166	170.1	3.88	0.9576	1.388	1.076	0.9998	-1.983	0.9995	0.9999	7.96	2.26	9.953	37.44	1
Range	23.39	2166	170.1	3.88	0.9578	1.387	1.076	0.8417	0.01686	0.9514	0.6363	5.96	2.259	9.953	37.44	0.295
Mean	6.306	558.1	47.96	0.9456	0.747	0.7771	0.4173	0.4315	-1.999	0.931	0.6246	3.808	1.396	2.656	9.677	0.8498
Std. Deviation	5.34	602.4	45.73	0.8572	0.1571	0.3446	0.259	0.2195	0.001752	0.1926	0.1649	1.415	0.5855	2.589	9.588	0.07591
Std. Error of M	0.3668	41.38	3.14	0.05887	0.01079	0.02367	0.01779	0.01508	0.0001203	0.01323	0.01133	0.09717	0.04021	0.1778	0.6585	0.005214
**Early Fibroatheroma**																
Minimum	1.001	0.006331	0.00294	0.0003271	0.2087	0.002951	0.0003271	0.152	-2	0.1153	0.3694	2.001	0.005368	0.0004589	0.001294	0.6911
25% Percentile	1.316	2.744	0.9079	0.06566	0.6674	0.2359	0.06242	0.2953	-2	0.8696	0.5219	2.22	0.516	0.1373	0.4586	0.8035
Median	2.252	40.08	5.394	0.1751	0.7769	0.4559	0.163	0.5326	-1.999	0.974	0.7157	2.773	1.027	0.4887	1.754	0.9207
75% Percentile	7.694	737.5	64.9	1.004	0.8578	0.9588	0.5064	0.7796	-1.999	0.9976	0.881	4.329	1.733	3.593	12.92	0.9692
Maximum	24.68	2102	177.3	3.695	0.9422	1.344	0.9562	0.9988	-1.993	0.9995	0.9994	8.034	2.256	10.23	37.54	0.9998
Range	23.68	2102	177.3	3.694	0.7335	1.341	0.9559	0.8468	0.006565	0.8842	0.6301	6.033	2.25	10.23	37.54	0.3088
Mean	5.133	414.5	34.49	0.5985	0.7422	0.5702	0.2849	0.5447	-1.999	0.8728	0.7028	3.388	1.1	1.99	7.359	0.8933
Std. Deviation	5.578	606.5	46.57	0.7716	0.1456	0.3857	0.2672	0.2651	0.0009816	0.2194	0.1931	1.52	0.6922	2.667	9.972	0.08426
Std. Error of M	0.4819	52.4	4.023	0.06666	0.01258	0.03332	0.02308	0.0229	0.0000848	0.01896	0.01668	0.1313	0.0598	0.2304	0.8615	0.007279
**Advancing Atheroma**																
Minimum	1.001	0.001845	-5.726	0.0003482	0.2696	0.003119	0.0003482	0.17	-2	0.09917	0.326	2.001	0.004081	0.0002677	0.0007228	0.6983
25% Percentile	2.339	73.39	8.861	0.2838	0.7486	0.5757	0.2226	0.2502	-1.999	0.9782	0.481	2.74	1.113	0.6324	2.22	0.7818
Median	5.559	611.3	57.45	0.8049	0.8121	0.8772	0.4392	0.3506	-1.999	0.996	0.5808	3.703	1.61	2.547	9.448	0.8325
75% Percentile	13.68	1483	99.14	1.958	0.8531	1.121	0.7086	0.4966	-1.999	0.9986	0.695	5.532	1.939	6.64	24.53	0.897
Maximum	30.43	2104	175.3	4.619	0.9304	1.405	1.174	0.9991	-1.99	0.9995	0.9996	9.083	2.223	10.96	41.52	0.9998
Range	29.43	2104	181	4.618	0.6609	1.402	1.174	0.8291	0.009584	0.9003	0.6736	7.082	2.219	10.96	41.52	0.3015
Mean	8.204	754	58.08	1.2	0.7828	0.82	0.4667	0.4154	-1.999	0.9566	0.6092	4.235	1.453	3.55	13	0.8436
Std. Deviation	6.979	692	48.85	1.079	0.1113	0.3536	0.2888	0.2087	0.0008453	0.1121	0.1627	1.761	0.5698	3.239	11.97	0.07618
Std. Error of M	0.4727	46.87	3.309	0.07307	0.007538	0.02395	0.01956	0.01413	0.00005725	0.007591	0.01102	0.1193	0.03859	0.2194	0.8107	0.00516
**K-means segmentation**																
**Early Fatty Streak Development**																
Minimum	1	0.0002409	0.0001645	0.000088	-0.0001554	0.0009097	0.000088	0.1581	-2	0.04815	0.3636	2	0.00103	0.0000536	0.0001262	0.705
25% Percentile	2.308	61.79	9.451	0.2747	0.7032	0.5609	0.2168	0.2752	-2	0.975	0.5064	2.726	1.065	0.6515	2.361	0.7905
Median	4.23	287.3	31.57	0.6642	0.7746	0.8288	0.3998	0.3731	-1.999	0.9941	0.5996	3.438	1.511	1.625	5.733	0.8429
75% Percentile	8.778	997.8	82.92	1.492	0.8447	1.049	0.6063	0.5235	-1.999	0.998	0.7124	4.633	1.846	4.414	16.34	0.8997
Maximum	24.39	2166	170.1	3.88	0.9576	1.388	1.076	0.9998	-1.983	0.9995	0.9999	7.96	2.26	9.953	37.44	1
Range	23.39	2166	170.1	3.88	0.9578	1.387	1.076	0.8417	0.01686	0.9514	0.6363	5.96	2.259	9.953	37.44	0.295
Mean	6.306	558.1	47.96	0.9456	0.747	0.7771	0.4173	0.4315	-1.999	0.931	0.6246	3.808	1.396	2.656	9.677	0.8498
Std. Deviation	5.34	602.4	45.73	0.8572	0.1571	0.3446	0.259	0.2195	0.001752	0.1926	0.1649	1.415	0.5855	2.589	9.588	0.07591
Std. Error of M	0.3668	41.38	3.14	0.05887	0.01079	0.02367	0.01779	0.01508	0.0001203	0.01323	0.01133	0.09717	0.04021	0.1778	0.6585	0.005214
**Early Fibroatheroma**																
Minimum	1.001	0.006331	0.00294	0.0003271	0.2087	0.002951	0.0003271	0.152	-2	0.1153	0.3694	2.001	0.005368	0.0004589	0.001294	0.6911
25% Percentile	1.316	2.744	0.9079	0.06566	0.6674	0.2359	0.06242	0.2953	-2	0.8696	0.5219	2.22	0.516	0.1373	0.4586	0.8035
Median	2.252	40.08	5.394	0.1751	0.7769	0.4559	0.163	0.5326	-1.999	0.974	0.7157	2.773	1.027	0.4887	1.754	0.9207
75% Percentile	7.694	737.5	64.9	1.004	0.8578	0.9588	0.5064	0.7796	-1.999	0.9976	0.881	4.329	1.733	3.593	12.92	0.9692
Maximum	24.68	2102	177.3	3.695	0.9422	1.344	0.9562	0.9988	-1.993	0.9995	0.9994	8.034	2.256	10.23	37.54	0.9998
Range	23.68	2102	177.3	3.694	0.7335	1.341	0.9559	0.8468	0.006565	0.8842	0.6301	6.033	2.25	10.23	37.54	0.3088

Development of an Image Classification Pipeline for Atherosclerotic Plaques Assessment using Supervised Machine Learning
Natasha N. Kunchur1, Leila B. Mostaço-Guidolin1

SUPPLEMENTARY MATERIAL

à **Descriptive statistics of FOS features, shape features and texture features**

Supplemental Table 3 Overview of the non-normalized descriptive statistics of FOS features, shape features and texture features

**Otsu Segmentation**																										
**Early Fatty Streak Development**																										
Mean	77500	0.08714	4452	0.4966	5.019	1	1.191	0.9603	0.4099	4772664	5.133	414.5	34.49	0.5985	0.7422	0.5702	0.2849	0.5447	-1.999	0.8728	0.7028	3.388	1.1	1.99	7.359	0.8933
Std. Deviation	41816	0.1041	2411	0.1504	0.762	0	1.345	2.538	0.1229	5132310	5.578	606.5	46.57	0.7716	0.1456	0.3857	0.2672	0.2651	0.0009816	0.2194	0.1931	1.52	0.6922	2.667	9.972	0.08426
Std. Error of M	3612	0.008989	208.3	0.01299	0.06583	0	0.1162	0.2192	0.01061	443364	0.4819	52.4	4.023	0.06666	0.01258	0.03332	0.02308	0.0229	0.0000848	0.01896	0.01668	0.1313	0.0598	0.2304	0.8615	0.007279
**Advancing Atheroma**																										
Minimum	195	0.002834	85.56	0.1224	2.804	1	0.009462	0.0000719	0.02726	10018	1.001	0.001845	-5.726	0.0003482	0.2696	0.003119	0.0003482	0.17	-2	0.09917	0.326	2.001	0.004081	0.0002677	0.0007228	0.6983
25% Percentile	26412	0.01456	2745	0.3421	4.434	1	0.02968	0.0003982	0.3051	1697173	2.339	73.39	8.861	0.2838	0.7486	0.5757	0.2226	0.2502	-1.999	0.9782	0.481	2.74	1.113	0.6324	2.22	0.7818
Median	68029	0.02402	5269	0.4333	4.848	1	0.1062	0.002638	0.4384	4434928	5.559	611.3	57.45	0.8049	0.8121	0.8772	0.4392	0.3506	-1.999	0.996	0.5808	3.703	1.61	2.547	9.448	0.8325
75% Percentile	94530	0.04631	7836	0.5186	5.214	1	0.5129	0.0378	0.4802	10534386	13.68	1483	99.14	1.958	0.8531	1.121	0.7086	0.4966	-1.999	0.9986	0.695	5.532	1.939	6.64	24.53	0.897
Maximum	156314	0.3709	14482	0.7483	6.682	1	4.129	12.29	0.5	32709684	30.43	2104	175.3	4.619	0.9304	1.405	1.174	0.9991	-1.99	0.9995	0.9996	9.083	2.223	10.96	41.52	0.9998
Range	156119	0.368	14397	0.6259	3.878	0	4.12	12.29	0.4727	32699666	29.43	2104	181	4.618	0.6609	1.402	1.174	0.8291	0.009584	0.9003	0.6736	7.082	2.219	10.96	41.52	0.3015
Mean	63554	0.04733	5467	0.4386	4.849	1	0.4753	0.2141	0.3754	7211275	8.204	754	58.08	1.2	0.7828	0.82	0.4667	0.4154	-1.999	0.9566	0.6092	4.235	1.453	3.55	13	0.8436
Std. Deviation	40248	0.06346	3185	0.1282	0.6449	0	0.7714	1.02	0.1405	7248439	6.979	692	48.85	1.079	0.1113	0.3536	0.2888	0.2087	0.0008453	0.1121	0.1627	1.761	0.5698	3.239	11.97	0.07618
Std. Error of M	2726	0.004298	215.7	0.008683	0.04368	0	0.05224	0.06907	0.009518	490926	0.4727	46.87	3.309	0.07307	0.007538	0.02395	0.01956	0.01413	0.00005725	0.007591	0.01102	0.1193	0.03859	0.2194	0.8107	0.00516
